# Supplementary material for: Reduced calcium levels and accumulation of abnormal insulin granules in stem cell models of HNF1A deficiency
Source: Commun Biol. 2022 Aug 2;5:779. doi: 10.1038/s42003-022-03696-z (PMC9345898; doi:10.1038/s42003-022-03696-z)
Supplement: Supplementary file 3 — Description of Additional Supplementary Files [file 42003_2022_3696_MOESM3_ESM.pdf]

Description of Additional Supplementary Files

File name: Supplementary Data 1
Description: Primer and oligo (sgRNA & ssDNA) sequence used for qPCR and CRISPR/Cas9 with HNF1A sgRNA off-target sites characterization.

File name: Supplementary Data 2
D**escription:** Sanger sequencing for HNF1A sgRNAs off-target genes in different cell lines.

File name: Supplementary Data 3
Description: List of down/up-regulated genes from bulk RNAseq transcriptome of INSGFP/wt sorted cells in vitro.

File name: Supplementary Data 4
Description: GOTERM and KEGG analysis from single cell RNAseq transcriptome of INSGFP/wt sorted cells in vitro.

File name: Supplementary Data 5
Description: GOTERM and KEGG analysis from single cell RNAseq transcriptome of unsorted SCisletlike cells in vitro.

File name: Supplementary Data 6
Description: Genotypes and ClinVar analysis of three HNF1A-MODY patients. HNF1A-MODY patient 2 (Pt2), mother of patient 3 (Pt3) and segregating for same HNF1A (+/R200Q) mutation had a history of gestational diabetes in 2 pregnancies and was diagnosed as having T2D in 2003 at age 38. She was managed with oral agents until 2015 for increasing hyperglycemia (292 mg/dl and HbA1c of 10.3%) when insulin was initiated and sulfonylurea discontinued. Since her last visit in 2016, fasting glucose levels remained elevated (216 mg/dl and HbA1c of 11.9%). HNF1AMODY patient 3 (Pt3) was misdiagnosed as having T1D at age 13 and was found to have a mutation in HNF1A (+/R200Q) at age 15. Insulin was discontinued and sulfonylurea treatment prescribed. Fasting glucose levels at age 19 were 177 mg/dl with an HbA1C of 7.8%, for which the sulfonylurea dosage was increased. Since then, Pt3's fasting glucose and HbA1C levels have risen progressively, reaching 338 mg/dl and 6.9% by 2017 at age 22. During the patient's last visit in 2017, non-fasting C-peptide levels were within the normal range (724 pM) but insulin was undetectable (0 pM).

File name: Supplementary Data 7
Description: Primary and secondary antibodies used for immunohistochemistry.

File name: Supplementary Data 8
Description: Source data.
