# Supplementary material for: Reduced calcium levels and accumulation of abnormal insulin granules in stem cell models of HNF1A deficiency
Source: Commun Biol. 2022 Aug 2;5:779. doi: 10.1038/s42003-022-03696-z (PMC9345898; doi:10.1038/s42003-022-03696-z)
Supplement: Supplementary file 12 — Reporting Summary [file 42003_2022_3696_MOESM12_ESM.pdf]

nature research

Corresponding author(s): Egli

Last updated by author(s): Apr 26, 2022

Reporting Summary

Nature Research wishes to improve the reproducibility of the work that we publish. This form provides structure for consistency and transparency in reporting. For further information on Nature Research policies, see our Editorial Policies and the Editorial Policy Checklist.

Statistics

For all statistical analyses, confirm that the following items are present in the figure legend, table legend, main text, or Methods section.

n/a	Confirmed	
☐	☒	The exact sample size (*n*) for each experimental group/condition, given as a discrete number and unit of measurement
☐	☒	A statement on whether measurements were taken from distinct samples or whether the same sample was measured repeatedly
☐	☒	The statistical test(s) used AND whether they are one- or two-sided<br>*Only common tests should be described solely by name; describe more complex techniques in the Methods section.*
☐	☒	A description of all covariates tested
☐	☒	A description of any assumptions or corrections, such as tests of normality and adjustment for multiple comparisons
☐	☒	A full description of the statistical parameters including central tendency (e.g. means) or other basic estimates (e.g. regression coefficient) AND variation (e.g. standard deviation) or associated estimates of uncertainty (e.g. confidence intervals)
☐	☒	For null hypothesis testing, the test statistic (e.g. *F*, *t*, *r*) with confidence intervals, effect sizes, degrees of freedom and *P* value noted<br>*Give P values as exact values whenever suitable.*
☐	☒	For Bayesian analysis, information on the choice of priors and Markov chain Monte Carlo settings
☐	☒	For hierarchical and complex designs, identification of the appropriate level for tests and full reporting of outcomes
☐	☒	Estimates of effect sizes (e.g. Cohen's *d*, Pearson's *r*), indicating how they were calculated

Our web collection on statistics for biologists contains articles on many of the points above.

Software and code

Policy information about availability of computer code

Data collection	n/a
Data analysis	n.a

For manuscripts utilizing custom algorithms or software that are central to the research but not yet described in published literature, software must be made available to editors and reviewers. We strongly encourage code deposition in a community repository (e.g. GitHub). See the Nature Research guidelines for submitting code & software for further information.

Data

Policy information about availability of data

All manuscripts must include a data availability statement. This statement should provide the following information, where applicable:

- Accession codes, unique identifiers, or web links for publicly available datasets
- A list of figures that have associated raw data
- A description of any restrictions on data availability

Single cell RNA sequencing and bulk RNA sequencing data were deposited in NCBI's Gene Expression Omnibus (GEO) database and accession number is GSE128331 and GSE129653.

Field-specific reporting

Please select the one below that is the best fit for your research. If you are not sure, read the appropriate sections before making your selection.

☒ Life sciences ☐ Behavioural & social sciences ☐ Ecological, evolutionary & environmental sciences

For a reference copy of the document with all sections, see nature.com/documents/nr-reporting-summary-flat.pdf

Life sciences study design

All studies must disclose on these points even when the disclosure is negative.

Sample size	sample size is indicated for each experiment where relevant.
Data exclusions	none
Replication	at least 3
Randomization	does not apply
Blinding	does not apply

Reporting for specific materials, systems and methods

We require information from authors about some types of materials, experimental systems and methods used in many studies. Here, indicate whether each material, system or method listed is relevant to your study. If you are not sure if a list item applies to your research, read the appropriate section before selecting a response.

Materials & experimental systems

n/a	Involved in the study	
☐	☒	Antibodies
☐	☒	Eukaryotic cell lines
☒	☐	Palaeontology and archaeology
☐	☒	Animals and other organisms
☐	☒	Human research participants
☒	☐	Clinical data
☒	☐	Dual use research of concern

Methods

n/a	Involved in the study	
☒	☐	ChIP-seq
☐	☒	Flow cytometry
☒	☐	MRI-based neuroimaging

Antibodies

Antibodies used	in Table S7
Validation	HNF1A knockout

Eukaryotic cell lines

Policy information about cell lines

Cell line source(s)	in house
Authentication	STR genotyping
Mycoplasma contamination	negative
Commonly misidentified lines (See ICLAC register)	does not apply

Animals and other organisms

Policy information about studies involving animals; ARRIVE guidelines recommended for reporting animal research

Laboratory animals	mus musculus

Wild animals | does not apply

Field-collected samples | does not apply

Ethics oversight | Columbia embryonic stem cell research oversight (ESCRO) and IRB (indicated in manuscript)

Note that full information on the approval of the study protocol must also be provided in the manuscript.

Human research participants

Policy information about studies involving human research participants

Population characteristics | HNF1A-MODY patients

Recruitment | word of mouth

Ethics oversight | COLUMBIA University ESCRO and IRB

Note that full information on the approval of the study protocol must also be provided in the manuscript.

Flow Cytometry

Plots

Confirm that:

☒ The axis labels state the marker and fluorochrome used (e.g. CD4-FITC).

☒ The axis scales are clearly visible. Include numbers along axes only for bottom left plot of group (a 'group' is an analysis of identical markers).

☒ All plots are contour plots with outliers or pseudocolor plots.

☒ A numerical value for number of cells or percentage (with statistics) is provided.

Methodology

Sample preparation | Clusters of endocrine cells were dissociated into single cells using TrypLE™ Express (Life Technology, 12605036). Cells were then fixed with 4% paraformaldehyde for 20 minutes at room temperature followed by 10 minutes permeabilization with cold methanol at -20ºC. Cells were washed with 3% donkey serum diluted in PBS and primary antibodies diluted (Table S7) in blocking solution containing 3% donkey serum with 0.1% triton X-100 diluted in PBS overnight at 4ºC.

Instrument | BD FACSAriaII

Software | FlowJo software

Cell population abundance | Pancreatic organoids are a mixture of cells
GFP was gated for RNA sequencing analysis.

Gating strategy | negative gating on cells stained with secondary antibody only

☒ Tick this box to confirm that a figure exemplifying the gating strategy is provided in the Supplementary Information.
