# Supplementary material for: Reduced calcium levels and accumulation of abnormal insulin granules in stem cell models of HNF1A deficiency
Source: Commun Biol. 2022 Aug 2;5:779. doi: 10.1038/s42003-022-03696-z (PMC9345898; doi:10.1038/s42003-022-03696-z)
Supplement: Supplementary file 2 — Supplementary Information [file 42003_2022_3696_MOESM2_ESM.pdf]

Supplementary Materials:

Fig. S1. Schematic overview of main figures.

Fig. S2. Generation of isogenic cell lines with *HNF1A* mutations in hESCs and HNF1A-MODY iPSCs.

Fig. S3. *HNF1A* is not required to generate pancreatic endocrine cells *in vitro*.

Fig. S4. Identification of thirteen stem cell-derived cell populations by single cell RNAseq *in vitro*.

Fig. S5. Identification of scβ- and scα-like-cells and their gene regulatory network *in vitro*.

Fig. S6. *HNF1A* deficiency results in a bias of endocrine cells towards the α-cell fate *in vitro.*

Fig. S7. *HNF1A* deficiency results in a bias of endocrine cells towards the α-cell fate *in vivo*.

Fig. S8. *HNF1A* deficiency affects insulin secretion in association with *CACNA1A* and *SYT13* down-regulation *in vitro*.

Fig. S9. *HNF1A* deficiency causes abnormal insulin granule structure.

Fig. S10. *HNF1A* deficient scβ-cells are unable to maintain glucose homeostasis in diabetic mice.

Fig. S11. *HNF1A* R200Q mutation is pathogenic and causes developmental bias towards the α-cell fate *in vitro.*

Fig. S12. HNF1A-MODY iPSC-derived β-cells are initially glucose responsive.

Fig. S13. *HNF1A* haploinsufficiency gradually impairs scβ-cell function *in vivo.*

Fig. S14. Western blot raw images

Supplemental Figures

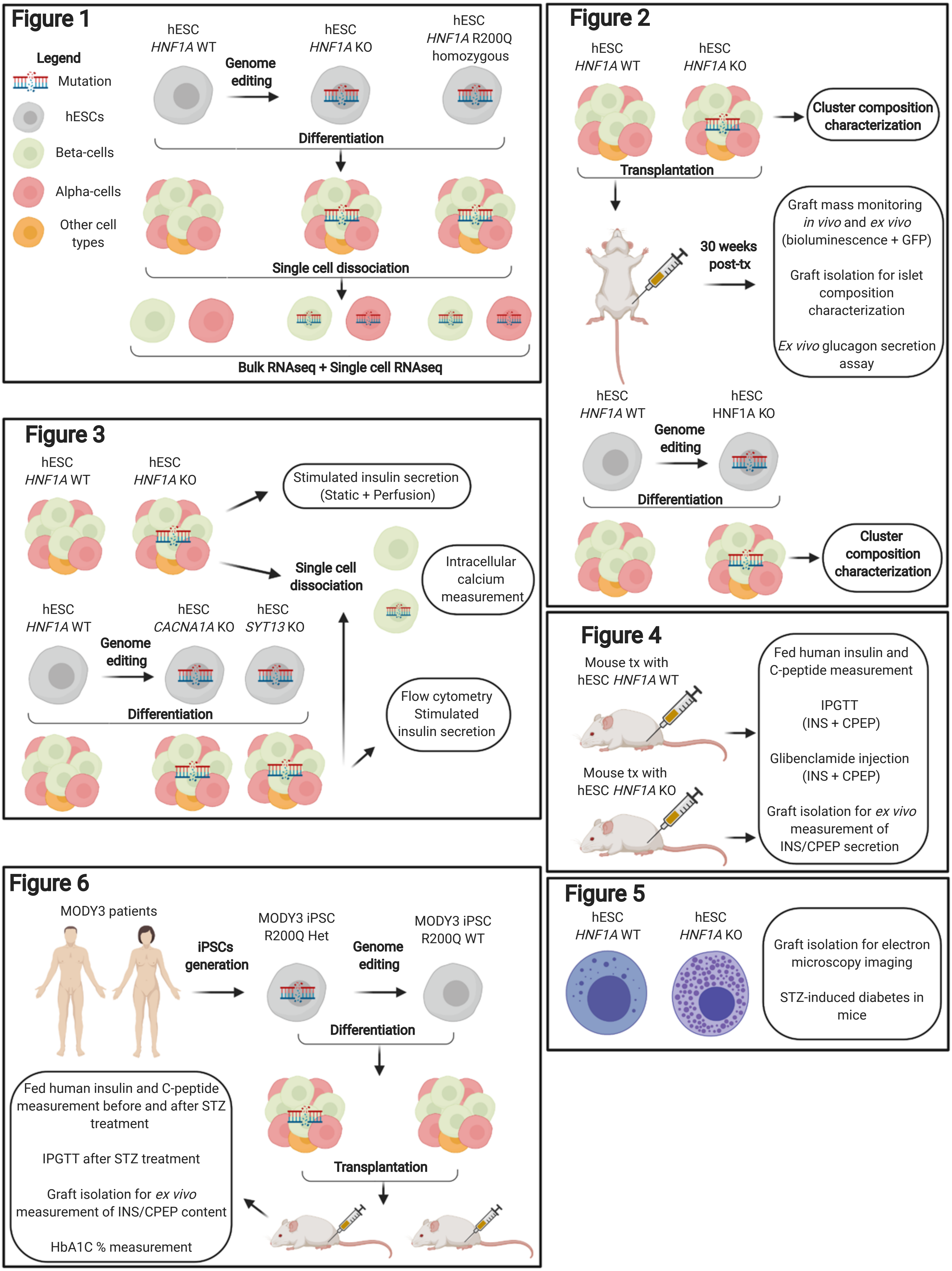
Figure 1
Legend
Mutation
hESCs
Beta-cells
Alpha-cells
Other cell types
hESC HNF1A WT
Genome editing
hESC HNF1A KO
hESC HNF1A R200Q homozygous
Differentiation
Single cell dissociation
Bulk RNAseq + Single cell RNAseq
Figure 2
hESC HNF1A WT
hESC HNF1A KO
Cluster composition characterization
Transplantation
30 weeks post-tx
Graft mass monitoring in vivo and ex vivo (bioluminescence + GFP)
Graft isolation for islet composition characterization
Ex vivo glucagon secretion assay
hESC HNF1A WT
hESC HNF1A KO
Genome editing
Differentiation
Cluster composition characterization
Figure 3
hESC HNF1A WT
hESC HNF1A KO
Stimulated insulin secretion (Static + Perfusion)
Single cell dissociation
Intracellular calcium measurement
hESC HNF1A WT
Genome editing
hESC CACNA1A KO
hESC SYT13 KO
Differentiation
Flow cytometry Stimulated insulin secretion
Figure 4
Mouse tx with hESC HNF1A WT
Mouse tx with hESC HNF1A KO
Fed human insulin and C-peptide measurement
IPGTT (INS + CPEP)
Glibenclamide injection (INS + CPEP)
Graft isolation for ex vivo measurement of INS/CPEP secretion
Figure 5
hESC HNF1A WT
hESC HNF1A KO
Graft isolation for electron microscopy imaging
STZ-induced diabetes in mice
Figure 6
MODY3 patients
iPSCs generation
MODY3 iPSC R200Q Het
Genome editing
MODY3 iPSC R200Q WT
Differentiation
Transplantation
Fed human insulin and C-peptide measurement before and after STZ treatment
IPGTT after STZ treatment
Graft isolation for ex vivo measurement of INS/CPEP content
HbA1C % measurement

Fig. S1. Schematic overview of main figures. IPGTT = intraperitoneal glucose tolerance test, STZ = streptozotocin, IHC = immunohistochemistry, MODY = maturity onset diabetes of the young, Het = heterozygous, WT = wild type. Created with BioRender.com by the authors.

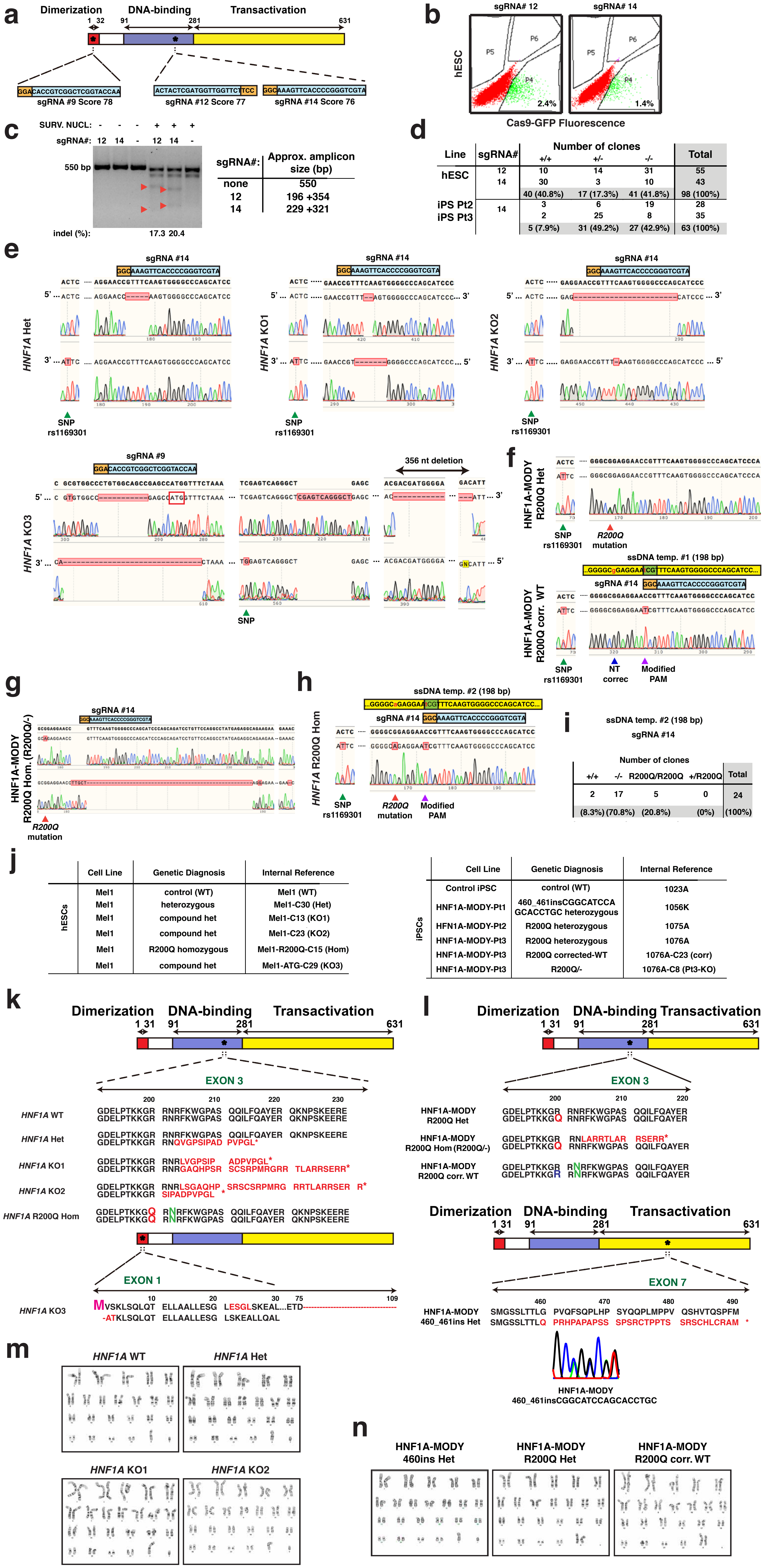
a
Dimerization
DNA-binding
Transactivation
1 32 91 281 631
sgRNA #9 Score 78
sgRNA #12 Score 77
sgRNA #14 Score 76
b
sgRNA# 12
sgRNA# 14
hESC
P5 P6 P4
2.4%
1.4%
Cas9-GFP Fluorescence
c
SURV. NUCL: - - - + + +
sgRNA#: 12 14 - 12 14 -
550 bp
indel (%): 17.3 20.4
sgRNA#: | Approx. amplicon size (bp)
none | 550
12 | 196 +354
14 | 229 +321
d
Line | sgRNA# | Number of clones +/+ | +/- | -/- | Total
hESC | 12 | 10 | 14 | 31 | 55
hESC | 14 | 30 | 3 | 10 | 43
| | 40 (40.8%) | 17 (17.3%) | 41 (41.8%) | 98 (100%)
iPS Pt2 | 14 | 3 | 6 | 19 | 28
iPS Pt3 | 14 | 2 | 25 | 8 | 35
| | 5 (7.9%) | 31 (49.2%) | 27 (42.9%) | 63 (100%)
e
sgRNA #14
HNF1A Het
HNF1A KO1
HNF1A KO2
SNP rs1169301
sgRNA #9
356 nt deletion
HNF1A KO3
SNP
f
HNF1A-MODY R200Q Het
SNP rs1169301
R200Q mutation
ssDNA temp. #1 (198 bp)
sgRNA #14
HNF1A-MODY R200Q corr. WT
NT correc
Modified PAM
g
sgRNA #14
HNF1A-MODY R200Q Hom. (R200Q/-)
R200Q mutation
h
ssDNA temp. #2 (198 bp)
sgRNA #14
HNF1A R200Q Hom
SNP rs1169301
R200Q mutation
Modified PAM
i
ssDNA temp. #2 (198 bp)
sgRNA #14
Number of clones: +/+ | -/- | R200Q/R200Q | +/R200Q | Total
2 | 17 | 5 | 0 | 24
(8.3%) | (70.8%) | (20.8%) | (0%) | (100%)
j
hESCs
Cell Line | Genetic Diagnosis | Internal Reference
Mel1 | control (WT) | Mel1 (WT)
Mel1 | heterozygous | Mel1-C30 (Het)
Mel1 | compound het | Mel1-C13 (KO1)
Mel1 | compound het | Mel1-C23 (KO2)
Mel1 | R200Q homozygous | Mel1-R200Q-C15 (Hom)
Mel1 | compound het | Mel1-ATG-C29 (KO3)
iPSCs
Cell Line | Genetic Diagnosis | Internal Reference
Control iPSC | control (WT) | 1023A
HNF1A-MODY-Pt1 | 460_461insCGGCATCCA GCACCTGC heterozygous | 1056K
HFN1A-MODY-Pt2 | R200Q heterozygous | 1075A
HNF1A-MODY-Pt3 | R200Q heterozygous | 1076A
HNF1A-MODY-Pt3 | R200Q corrected-WT | 1076A-C23 (corr)
HNF1A-MODY-Pt3 | R200Q/- | 1076A-C8 (Pt3-KO)
k
Dimerization
DNA-binding
Transactivation
1 31 91 281 631
EXON 3
HNF1A WT
HNF1A Het
HNF1A KO1
HNF1A KO2
HNF1A R200Q Hom
EXON 1
HNF1A KO3
l
Dimerization
DNA-binding
Transactivation
EXON 3
HNF1A-MODY R200Q Het
HNF1A-MODY R200Q Hom (R200Q/-)
HNF1A-MODY R200Q corr. WT
EXON 7
HNF1A-MODY 460_461ins Het
HNF1A-MODY 460_461insCGGCATCCAGCACCTGC
m
HNF1A WT
HNF1A Het
HNF1A KO1
HNF1A KO2
n
HNF1A-MODY 460ins Het
HNF1A-MODY R200Q Het
HNF1A-MODY R200Q corr. WT

Fig. S2. Generation of isogenic cell lines with *HNF1A* mutations in hESCs and HNF1A-MODY iPSCs. (a) Diagram illustrating functional domains of HNF1A protein with sgRNAs #12 (on-target score 77) and #14 (on-target score 76) targeting the DNA-binding domain (exon 3). **(b)** FACs sorting of hESCs nucleofected with Cas9-GFP and sgRNA#12 or #14 with 2.4% and 1.4% trasnfection efficiency. **(c)** Surveyor assay to assess sgRNA indel efficiency in *HNF1A* gene. *HNF1A* PCR products with (+) or without (-) surveyor nuclease or sgRNA indicating cutting efficiency of 17.3% and 20.4% for sgRNA#12 and sgRNA#14, respectively, with indicated amplicon sizes (Table). The surveyor assay cleaves DNA at the site of heterozygosity. Bands with amplicon size of ~500bp are due to the linked heterozygous SNPs rs1169301, creating cleavage. **(d)** Table showing the percentage of mutant lines generated after CRISPR/Cas9 in hESCs leading to 17.3% heterozygous clones (17 clones out of 98 total) and 41.8% compound heterozygous clones (41 clones out of 98 total), indicating a 59.2% of mutation efficiency (58 clones out of 98 total). 4 clones of the 98 (4%) appeared homozygous by Sanger sequencing, but also lacked two linked SNPs, rs1169301 (102 bp from PAM sequence of sgRNAs#14 to SNP) and rs2071190 (130 bp from PAM sequence of sgRNAs#14 to SNP), which are both located in the second intron. These homozygous lines (4/98, 4%) could be caused by a large deletion, and were not used for further studies. In HNF1A-MODY iPSC, 92.1% of clones were (+/-, heterozygous) and (-/-, compound heterozygous). Mutation-correction efficiency of HNF1A-MODY iPSC Pt2/Pt3 was 7.9% (+/+, wild type) from total number of clones. **(e)** Sanger sequencing results showing heterozygous (Het) and knockout frameshift mutations (KO1 and KO2) from both alleles in exon3 and knockout frameshift mutations (KO3) in exon1 near the start codon (red box). **(f)** Sanger sequencing showing

heterozygous R200Q mutation (red arrow) in HNF1A-MODY iPSC line, after correction of the mutation (blue arrow) in HNF1A-MODY iPSC line (HNF1A-MODY iPSC R200Q-corrected WT) using a ssDNA template donor #1 (198 bp). **(g)** Sanger sequencing after introduction of a frameshift mutations in the wild type allele of HNF1A-MODY iPSC line (R200Q/-). **(h)** Sanger sequencing results showing R200Q homozygous mutation introduction (red arrow) in hESCs using a ssDNA template donor #2 (198 bp). PAM sequence in template donor was modified (C>T, silent mutation) to increase recombination efficiency (purple arrow). Heterozygosity at the upstream rs1169301 SNP C>T in *HNF1A* (green arrow; 102 bp from PAM sequence) indicates that both alleles are detected. All Sanger sequencing results were verified by TOPO® cloning of at least six clones per genotype. **(i)** Table showing the percentage of clones with R200Q homozygous mutation generated (20.8%) in hESCs from total number of clones. **(j)** Genetic information of cell lines (hESCs and iPSCs) included in the study**. (k)** Frameshift mutations in exon 3 of hESC cell lines leading to premature stop-codons (*) and generation of truncated versions of HNF1A protein (different amino acids from WT in red). For *HNF1A* KO3, mutation is at start codon in exon 1, indicated in red are different amino acids from WT sequence, red dash are deletions and in magenta the start codon. **(l)** Mutations in exon 3 of iPSC cell lines leading to heterozygous amino acid substitution (R200Q heterozygous), generation of R200Q homozygous (R200Q/-) and isogenic R200Q-corrected WT lines (+/+). For HNF1A-MODY iPSC 460_461ins Het line (ID#1056), CGGCATCCAGCACCTGC insertion is in exon 7 as shown by Sanger Sequencing, leading to frameshift mutations and premature stop codon (*). Different amino acids from WT in red, amino acid correction in bleu and amino acid silent mutation in green. **(m)** Cytogenic analysis performed on 20 G-banded metaphase cells from hESC and **(n)** iPSC lines indicating normal karyotypes.

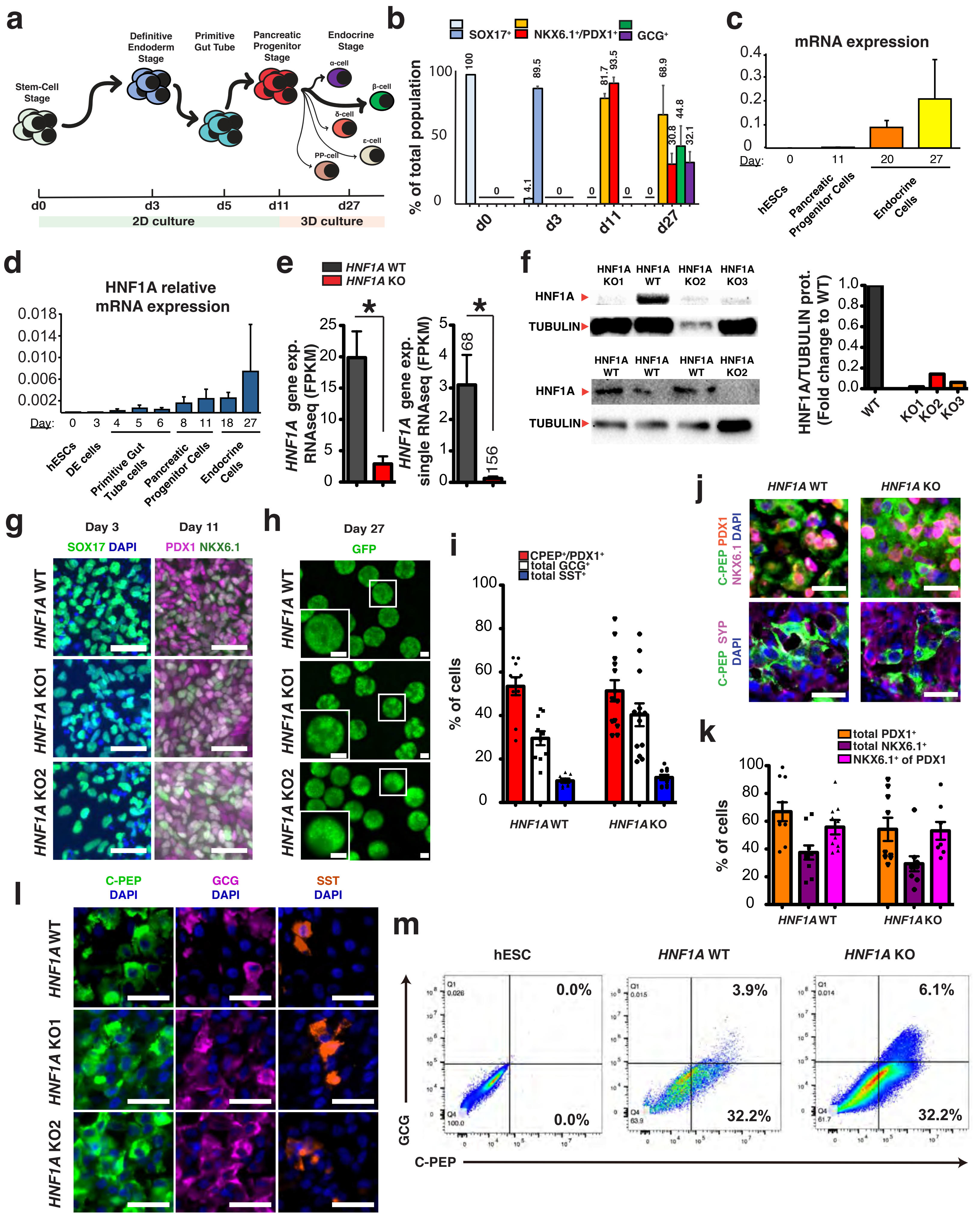

a
Stem-Cell Stage
Definitive Endoderm Stage
Primitive Gut Tube
Pancreatic Progenitor Stage
Endocrine Stage
α-cell
β-cell
δ-cell
ε-cell
PP-cell
d0
d3
d5
d11
d27
2D culture
3D culture
b
SOX17+
NKX6.1+/PDX1+
GCG+
% of total population
c
mRNA expression
Day:
hESCs
Pancreatic Progenitor Cells
Endocrine Cells
d
HNF1A relative mRNA expression
Day:
hESCs
DE cells
Primitive Gut Tube cells
Pancreatic Progenitor Cells
Endocrine Cells
e
HNF1A WT
HNF1A KO
HNF1A gene exp. RNAseq (FPKM)
HNF1A gene exp. single RNAseq (FPKM)
f
HNF1A KO1
HNF1A WT
HNF1A KO2
HNF1A KO3
HNF1A
TUBULIN
HNF1A WT
HNF1A KO2
HNF1A/TUBULIN prot. (Fold change to WT)
WT
KO1
KO2
KO3
g
Day 3
SOX17 DAPI
Day 11
PDX1 NKX6.1
HNF1A WT
HNF1A KO1
HNF1A KO2
h
Day 27
GFP
i
CPEP+/PDX1+
total GCG+
total SST+
% of cells
HNF1A WT
HNF1A KO
j
HNF1A WT
HNF1A KO
C-PEP PDX1 NKX6.1 DAPI
C-PEP SYP DAPI
k
total PDX1+
total NKX6.1+
NKX6.1+ of PDX1
% of cells
HNF1A WT
HNF1A KO
l
C-PEP DAPI
GCG DAPI
SST DAPI
HNF1A WT
HNF1A KO1
HNF1A KO2
m
hESC
HNF1A WT
HNF1A KO
0.0%
0.0%
3.9%
32.2%
6.1%
32.2%
GCG
C-PEP

Fig. S3. *HNF1A* is not required to generate pancreatic endocrine cells *in vitro*. (a) Overview of the different stages of stem cell differentiation to pancreatic progenitor cells. The initial stages of differentiation were conducted in planar culture from day 0 to day 11 followed by 3D clustering on day 12 to form aggregates or clusters of endocrine cells on day 27. **(b)** Percentage of total cell population by immunohistochemistry (IHC) expressing key markers of pluripotency stage (OCT4, d0), definitive endoderm stage (SOX17, d3), pancreatic progenitor stage (PDX1 and NKX6.1, d11) and endocrine stage (CPEP, c-peptide; GCG, glucagon, d27). **(c)** *INS* and **(d)** *HNF1A* relative mRNA expression levels determined by qPCR throughout the differentiation from hESC *HNF1A* WT cells (n=3-7). Each independent biological replicate (n) consists of 2-3 technical replicates for all experiments. **(e)** *HNF1A* gene expression levels (FPKM) determined by RNAseq after FACs sorting of $INS^{GFP/wt}$ positive cells from hESC-derived endocrine cells (WT n=3 and KO n=3) on day 27 of differentiation *in vitro*. Numbers on top of histogram denotes total number of single cells used for analysis. **(f)** Western blot for HNF1A (67 kDa) and β-Tubulin-III (55 kDa) from hESC-derived endocrine cells with respective quantification on day 27 of differentiation *in vitro*. **(g)** Representative IHC staining of hESC-derived cells *in vitro* at definitive endoderm stage with SOX17 (day 3) and pancreatic progenitor stage with PDX1 and NKX6.1 (day 11). Scale bars: 50 µm. White cells are PDX1/NKX6.1 double positive cells. **(h)** Representative GFP images. Scale bars at 200 µm. **(i)** Percentage of scβ-like-cells ($CPEP^+/PDX1^+$ in red), scα-like-cells (GCG^+ in white) and scδ-like-cells (SST^+ in blue) by IHC (day 27). **(j)** Representative IHC image of hESC-derived endocrine cell lines for indicated markers with **(k)** quantification of $PDX1^+$ and $NKX6.1^+$ cells (day 27). (**l**) Representative IHC image of hESC-derived endocrine cell lines for indicated markers. (**m**) $CPEP^+$ and GCG^+ populations by flow cytometry for hESC, WT and KO at day 27 of differentiation *in vitro*. Gating for GCG and CPEP negative cells was determined by incubating

hESC cells (negative control) with primary and with secondary antibodies. Scale bars: 20 µm. 20 clusters (~10k cells per cluster) of endocrine cells were used flow cytometry. For scatter plots, each point in plots represents an independent biological experiment (n). Data are represented as mean ± SEM. p-values: *$p<0.05$, **$p<0.01$, ***$p<0.001$, two-tailed t-test.

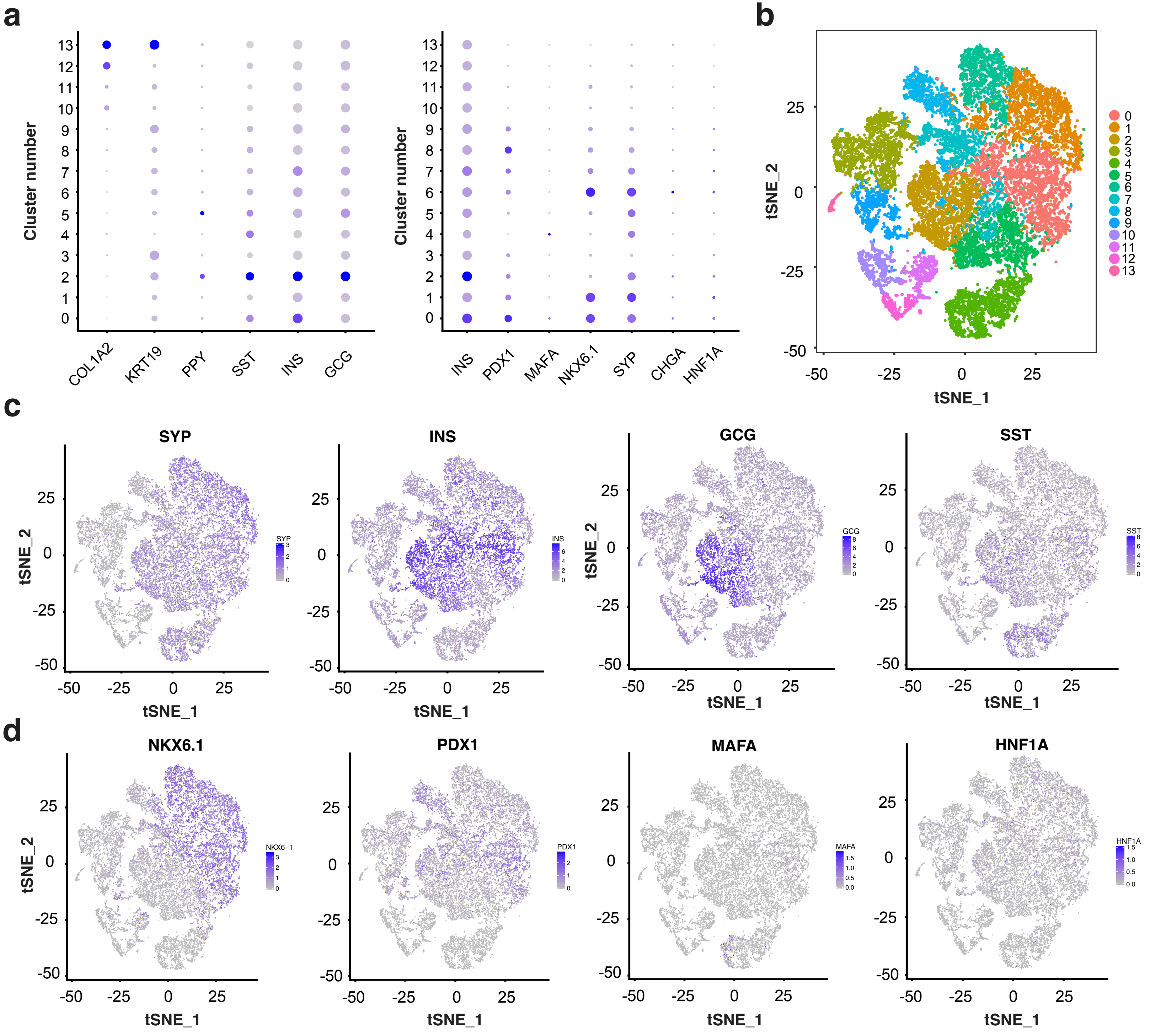
a
Cluster number
13
12
11
10
9
8
7
6
5
4
3
2
1
0
COL1A2
KRT19
PPY
SST
INS
GCG
INS
PDX1
MAFA
NKX6.1
SYP
CHGA
HNF1A
b
tSNE_2
tSNE_1
0
1
2
3
4
5
6
7
8
9
10
11
12
13
c
SYP
INS
GCG
SST
d
NKX6.1
PDX1
MAFA
HNF1A

Fig. S4. Identification of thirteen stem cell-derived cell populations by single cell RNAseq *in vitro*. (a-d) Single cell RNA sequencing of 22164 (all genotypes combined) unsorted hESC-derived endocrine cells (n=3 for each genotype) *in vitro*. **(a)** Cell type clustering of cells based on their normalized expression of different pancreatic markers: *COL1A2* (pancreatic stellate cell), *KRT19* (ductal cell), *PPY* (γ-cell), *SST* (δ-cell), *INS* (β-cell), and *GCG* (α-cell). A total of 13 clusters were identified. Size is the percentage of cells expressing the indicated marker and color intensity the average expression level of the indicated marker, where blue indicates the highest expression level and gray the lowest. **(b)** Feature plot based on tSNE projection of cells where the colors denote different cell type clusters identified (total of 13 identified) by *HNF1A* genotype line via Louvain algorithm performed by Seurat. **(c)** Feature plot based on tSNE projection of cells based on the normalized expression of endocrine markers *SYP* (synaptophysin), *INS* (insulin), *GCG* (glucagon) and *SST* (somatostatin) and **(d)** pancreatic transcription factors (*NKX6.1*, *PDX1*, *MAFA* and *HNF1A*) where blue indicates the highest expression level and gray the lowest. Genes expressed at low levels will have fewer labeled cells because of dropout. All stem cell differentiations were done for 27-30 days.

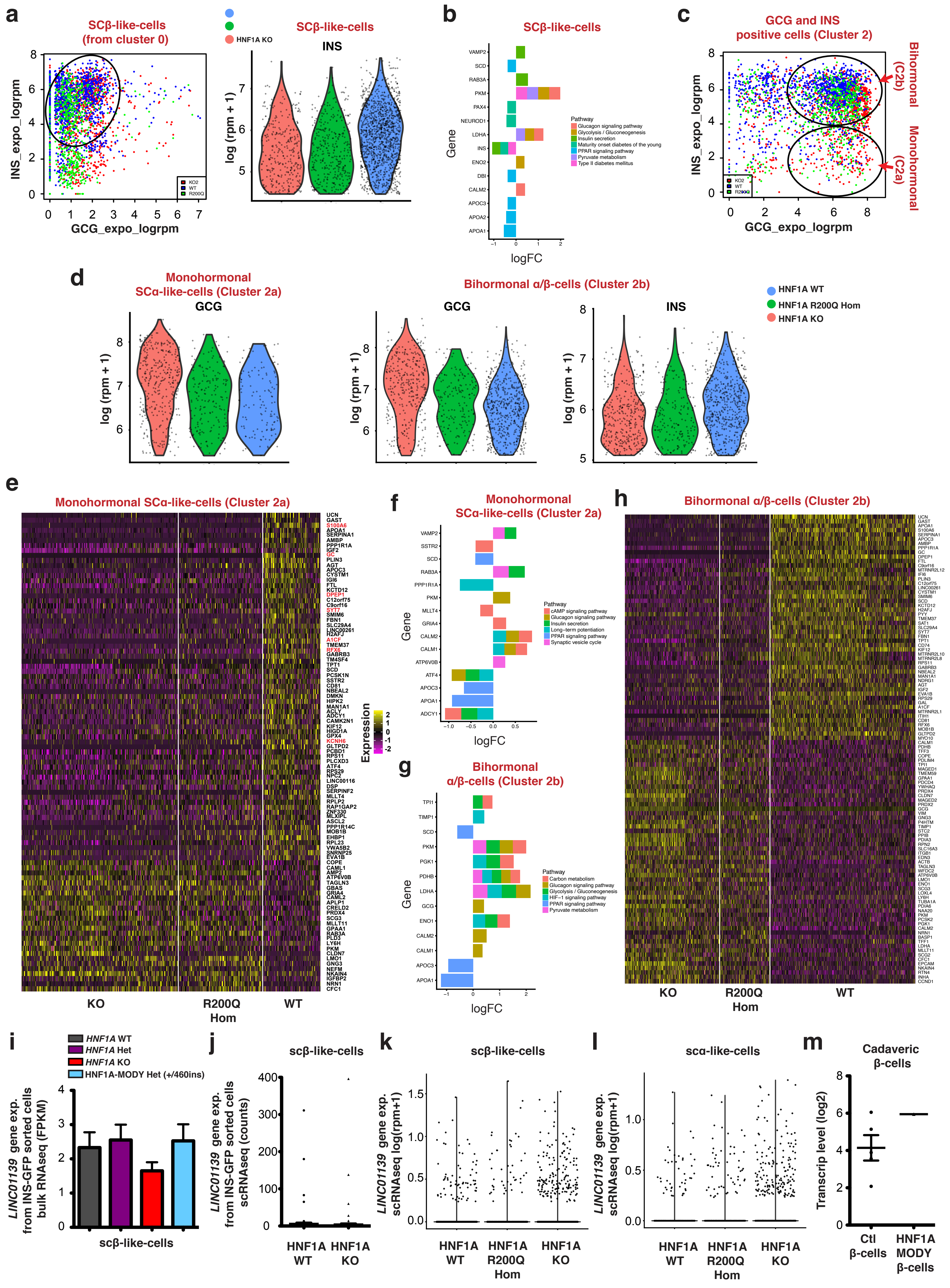
a
SCβ-like-cells (from cluster 0)
INS_expo_logrpm
GCG_expo_logrpm
KO2
WT
R200Q
SCβ-like-cells
HNF1A KO
INS
log (rpm + 1)
b
SCβ-like-cells
VAMP2
SCD
RAB3A
PKM
PAX4
NEUROD1
LDHA
INS
ENO2
DBI
CALM2
APOC3
APOA2
APOA1
Gene
logFC
Pathway
Glucagon signaling pathway
Glycolysis / Gluconeogenesis
Insulin secretion
Maturity onset diabetes of the young
PPAR signaling pathway
Pyruvate metabolism
Type II diabetes mellitus
c
GCG and INS positive cells (Cluster 2)
INS_expo_logrpm
GCG_expo_logrpm
KO2
WT
R200Q
Bihormonal (C2b)
Monohormonal (C2a)
d
Monohormonal SCα-like-cells (Cluster 2a)
GCG
log (rpm + 1)
Bihormonal α/β-cells (Cluster 2b)
GCG
INS
HNF1A WT
HNF1A R200Q Hom
HNF1A KO
e
Monohormonal SCα-like-cells (Cluster 2a)
UCN
GAST
S100A6
APOA1
SERPINA1
AMBP
PPP1R1A
IGF2
GC
PLIN3
AGT
APOC3
CYSTM1
IGI6
FTL
KCTD12
DPEP1
C12orf75
C9orf16
SYT7
SMIM6
FBN1
SLC29A4
LINC00261
H2AFJ
A1CF
TMEM37
RFX6
GABRB3
TM4SF4
TPT1
SCD
PCSK1N
SSTR2
CD81
NBEAL2
DMKN
HIPK2
MAN1A1
ACLY
ADCY1
CAMK2N1
KIF12
HIGD1A
GPX4
KCNH6
GLTPD2
PCBD1
RPS11
PLCXD3
ATF4
RPS29
NPC2
LINC00116
DSP
SERPINF2
MLLT4
RPLP2
RAP1GAP2
ZNF330
MLXIPL
ASCL2
PPP1R14C
MOB1B
EHBP1
RPL23
VWA5B2
SNRNP25
EVA1B
COPE
CAML1
AMP2
ATP6V0B
TAGLN3
GBAS
CAML2
APLP1
CRELD2
PRDX4
SCG3
MLLT11
GPAA1
RAB3A
PLD3
LY6H
PKM
CLDN7
LMO1
GNG3
NEFM
NKAIN4
IGFBP2
NRN1
CFC1
Expression
KO
R200Q Hom
WT
f
Monohormonal SCα-like-cells (Cluster 2a)
VAMP2
SSTR2
SCD
RAB3A
PPP1R1A
PKM
MLLT4
GRIA4
CALM2
CALM1
ATP6V0B
ATF4
APOC3
APOA1
ADCY1
Gene
logFC
Pathway
cAMP signaling pathway
Glucagon signaling pathway
Insulin secretion
Long−term potentiation
PPAR signaling pathway
Synaptic vesicle cycle
g
Bihormonal α/β-cells (Cluster 2b)
TPI1
TIMP1
SCD
PKM
PGK1
PDHB
LDHA
GCG
ENO1
CALM2
CALM1
APOC3
APOA1
Gene
logFC
Pathway
Carbon metabolism
Glucagon signaling pathway
Glycolysis / Gluconeogenesis
HIF−1 signaling pathway
PPAR signaling pathway
Pyruvate metabolism
h
Bihormonal α/β-cells (Cluster 2b)
KO
R200Q Hom
WT
i
HNF1A WT
HNF1A Het
HNF1A KO
HNF1A-MODY Het (+/460ins)
LINC01139 gene exp. from INS-GFP sorted cells bulk RNAseq (FPKM)
scβ-like-cells
j
scβ-like-cells
LINC01139 gene exp. from INS-GFP sorted cells scRNAseq (counts)
HNF1A WT
HNF1A KO
k
scβ-like-cells
LINC01139 gene exp. scRNAseq log(rpm+1)
HNF1A WT
HNF1A R200Q Hom
HNF1A KO
l
scα-like-cells
LINC01139 gene exp. scRNAseq log(rpm+1)
HNF1A WT
HNF1A R200Q Hom
HNF1A KO
m
Cadaveric β-cells
Transcrip level (log2)
Ctl β-cells
HNF1A MODY β-cells

Fig. S5. Identification of scβ- and scα-like-cells and their gene regulatory network *in vitro*. **(a)** Scatter plot of 3133 hESC-derived β-like-cells from cluster 0 displayed (**Fig. S4**) based on *GCG* and *INS* expression (expo_logrpm). Total cell number from all genotypes combined. Encircled are scβ-like-cells (total of 1846 cells) with violin plot displayed based on *INS* expression (log1(rpm+1)). **(b)** Barplot showing genes differentially expressed as log2FC and enriched in specific pathway from scβ-like-cells. **(c)** Scatter plot of 2670 hESC-derived α-like-cells from cluster 2 (**Fig. S4**) displayed based on *GCG* and *INS* expression (expo_logrpm). Total cell number from all genotypes combined. Encircled are monohormonal scα-like-cells (Cluster 2a) (total of 641 cells) and bihormonal scα-like-cells (Cluster 2b) (total of 1054 cells). **(d)** Violin plot of indicated cluster of cells displayed based on *GCG* and *INS* expression (log1(rpm+1)). Monohormonal scβ-like-cells (cluster 0) and bihormonal scα-like-cells (cluster 2b) were identified and displayed as |logFC|>0.35 and adjusted p-value $<1e^{-4}$. Monohormonal scα-like-cells (cluster 2a) displayed as |logFC|>0.25 and adjusted p-value $<1e^{-2}$. **(e)** Heatmap showing differentially expressed genes from monohormonal scα-like-cells (Cluster 2a) by *HNF1A* genotypes. Total of 641 scα-like-cells (cluster 2a) (all genotypes combined) were identified and displayed as |logFC|>0.25 and adjusted p-value $<1e^{-2}$. **(f and g)** Barplot showing genes differentially expressed as log2FC and enriched in specific pathway from indicated endocrine cell type. **(h)** Heatmap showing top differentially expressed genes from bihormonal α/β-like-cells (Cluster 2b) across genotypes. Genes are listed in decreasing order of $\log_2$ fold change between *HNF1A* WT and *HNF1A* mutant genotypes. n=3 for each genotype. **(i-m)** Gene expression profile of *LINC01139* in scβ-like-cells, scα-like-cells and cadaveric β-cells. **(i)** *LINC01139* gene expression levels (FPKM) determined by bulk RNAseq and after FACs sorting of $INS^{GFP/wt}$ positive cells from

hESC-derived endocrine cells (hESC WT n=3, hESC Het n=3, hESC KO n=3 and HNF1A-MODY iPSC 460ins Het n=2). **(j)** *HNF1A* gene expression levels (FPKM) determined by single-cell RNAseq after FACs sorting of $INS^{GFP/wt}$ positive cells from hESC-derived endocrine cells (hESC WT n=113 cells and hESC KO n=158 cells). **(k)** Violin plot of scβ-like-cells and **(l)** scα-like-cells based on *LINC01139* expression (log1(rpm+1)) by genotype. **(m)** *LINC01139* transcript levels of sorted pancreatic β-cells from non-diabetic donors (n=5) and HNF1A-MODY donor (n=1) (Haliyur et al. 2019). All stem cell differentiations were done for 27 days.

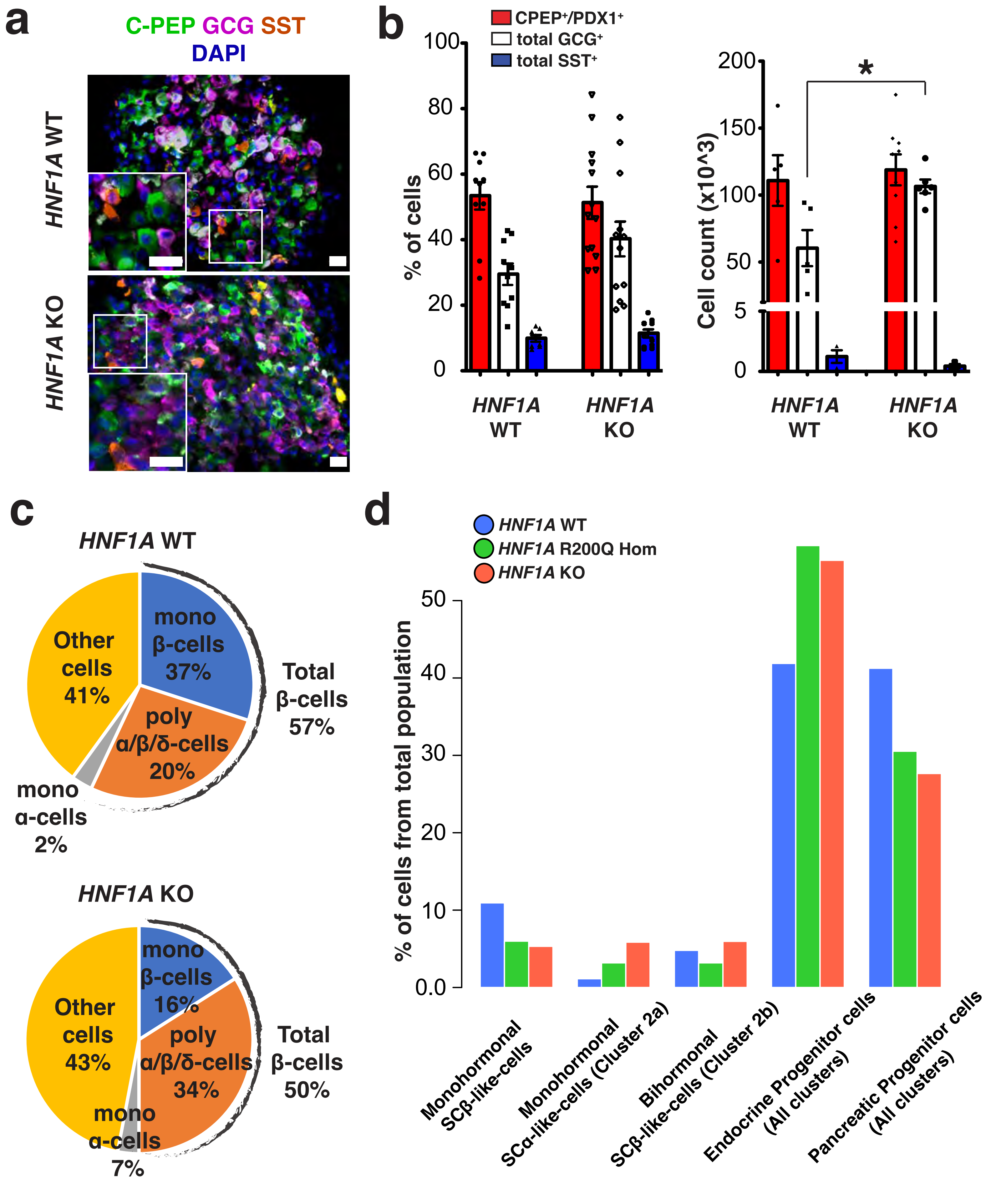
a
C-PEP GCG SST
DAPI
HNF1A WT
HNF1A KO
b
CPEP+/PDX1+
total GCG+
total SST+
% of cells
Cell count (x10^3)
*
HNF1A WT
HNF1A KO
c
HNF1A WT
mono β-cells 37%
poly α/β/δ-cells 20%
mono α-cells 2%
Other cells 41%
Total β-cells 57%
HNF1A KO
mono β-cells 16%
poly α/β/δ-cells 34%
mono α-cells 7%
Other cells 43%
Total β-cells 50%
d
HNF1A WT
HNF1A R200Q Hom
HNF1A KO
% of cells from total population
Monohormonal SCβ-like-cells
Monohormonal SCα-like-cells (Cluster 2a)
Bihormonal SCβ-like-cells (Cluster 2b)
Endocrine Progenitor cells (All clusters)
Pancreatic Progenitor cells (All clusters)

Fig. S6. *HNF1A* deficiency causes a developmental bias towards the α-cell fate *in vitro.* (a) Representative IHC images of hESC-derived endocrine cell lines with indicated markers and **(b)** quantification of percentage and total cell count of scβ-like-cells ($CPEP^+$/$PDX1^+$ in red), scα-like-cells (GCG^+ in white) and scδ-like-cells (SST^+ in blue). Cell count performed from 20 clusters. **(c)** Endocrine cells as percentage from total cells from hESC-derived clusters based on IHC staining. Monohormonal β-cells are $CPEP^+$/GCG^-/SST^- cells and polyhormonal α/β/δ-cells are $CPEP^+$/GCG^+/SST^+ cells. **(d)** Barplot showing the percentage (%) of indicated endocrine cell types from total cells (22164 cells) analyzed by single cell RNAseq from hESC-derived endocrine cells by *HNF1A* genotype (n=3 for each genotype). Endocrine progenitor cells are *SYP* expressing cells and pancreatic progenitor cells are *PDX1* expressing cells. All stem cell differentiations were done for 27 days. For scatter plots, each point in plots represents an independent biological experiment (n). Data are represented as mean ± SEM. p-values: *p<0.05, **p<0.01, ***p<0.001; Mann-Whitney test. n.s: non-significant. Scale bars: 20 µm.

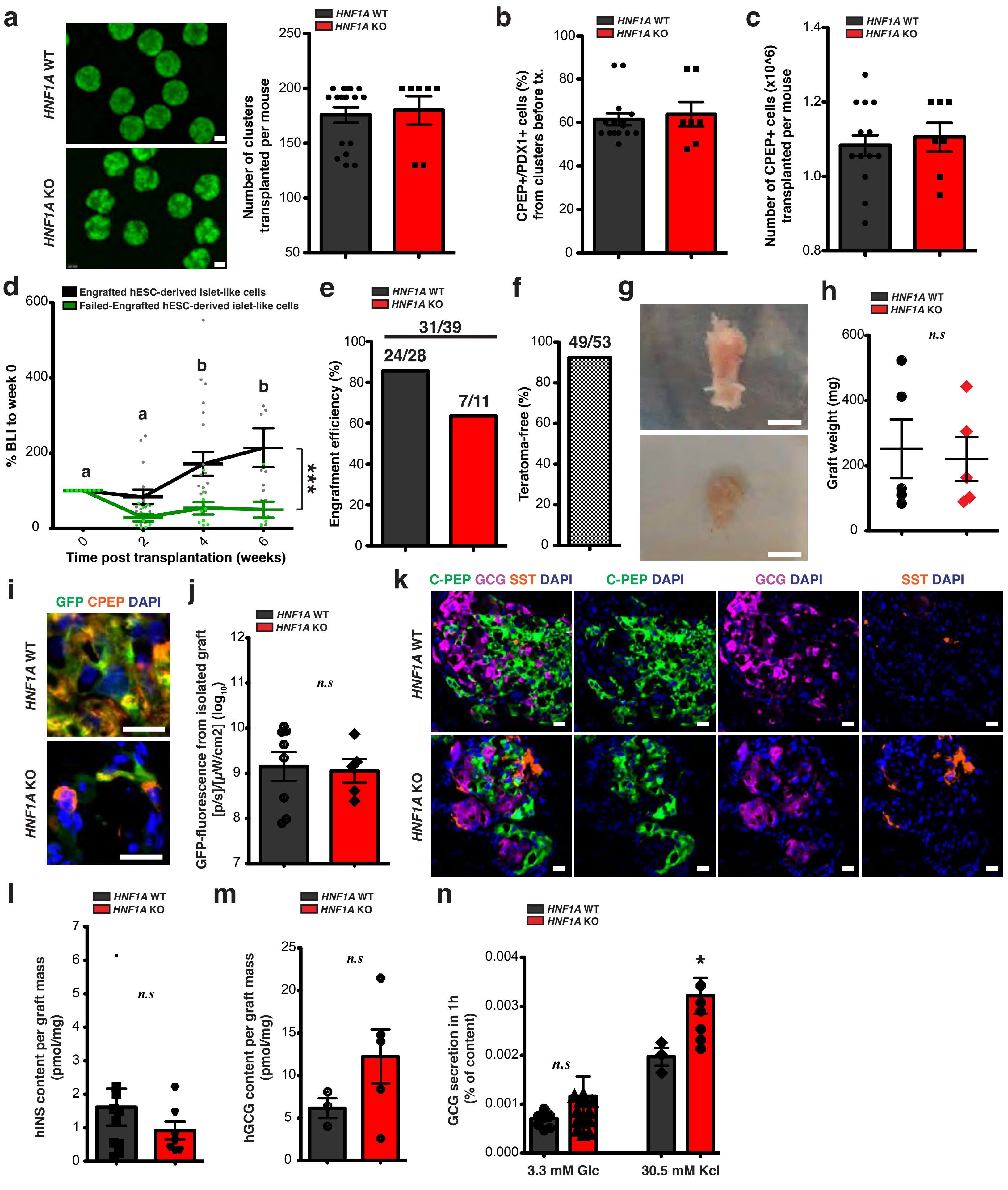

a
HNF1A WT
HNF1A KO
HNF1A WT
HNF1A KO
Number of clusters transplanted per mouse
b
HNF1A WT
HNF1A KO
CPEP+/PDX1+ cells (%) from clusters before tx.
c
HNF1A WT
HNF1A KO
Number of CPEP+ cells (x10^6) transplanted per mouse
d
Engrafted hESC-derived islet-like cells
Failed-Engrafted hESC-derived islet-like cells
% BLI to week 0
Time post transplantation (weeks)
e
HNF1A WT
HNF1A KO
31/39
24/28
7/11
Engrafment efficiency (%)
f
49/53
Teratoma-free (%)
g
h
HNF1A WT
HNF1A KO
n.s
Graft weight (mg)
i
GFP CPEP DAPI
HNF1A WT
HNF1A KO
j
HNF1A WT
HNF1A KO
n.s
GFP-fluorescence from isolated graft [p/s]/[μW/cm2] (log10)
k
C-PEP GCG SST DAPI
C-PEP DAPI
GCG DAPI
SST DAPI
HNF1A WT
HNF1A KO
l
HNF1A WT
HNF1A KO
n.s
hINS content per graft mass (pmol/mg)
m
HNF1A WT
HNF1A KO
n.s
hGCG content per graft mass (pmol/mg)
n
HNF1A WT
HNF1A KO
n.s
GCG secretion in 1h (% of content)
3.3 mM Glc
30.5 mM Kcl

Fig. S7. *HNF1A* deficiency causes a developmental bias towards the α-cell fate *in vivo* (a) Representative image and number (WT n=17 and KO n=7) of pancreatic islet-like clusters transplanted per mouse and per genotype. Scale bars at 200 µm. **(b)** Percentage (%) of $CPEP^{+}/PDX1^{+}$ cells from clusters before transplantation with **(c)** total number of $CPEP^{+}$ cells (10x6) transplanted per mouse and per genotype. **(d)** % Bioluminescence intensity (BLI) normalized to week 0 (transplantation day) of mice transplanted with *GAPDH*$^{Luciferase/wt}$ reporter hESC lines 0, 2, 4 and 6 weeks post-transplantation. Engrafted hESC-derived islet-like cells (WT n=11 and KO n=4) resulted in >50 pM of circulating human c-peptide after 6 weeks. Non-engrafted hESC-derived islet-like cells (WT n=4 and KO n=6) resulted in no or <5 pM of circulating human c-peptide after 30 weeks. Results are presented as mean of mice transplanted with both hESC genotypes. p-values were b: $p<0.05$. **(e)** Engraftment efficiency (%) from all mice transplanted with islet-like cells. **(f)** Teratoma-free mice transplanted with hESC-derived islet-like cell lines. Graft tissue was considered a teratoma when larger than 2 cm and by IHC of the germ layers. **(g)** Representative image of graft tissue 30 weeks post-transplantation with hESC-derived endocrine cells after explant from quadriceps muscle. Scale bars: 0.5 cm. **(h)** Graft weight in mg from explants. **(i)** Representative IHC image from isolated grafts for indicated markers. Scale bars: 20 µm. **(j)** GFP fluorescence ($[p/s]/[\mu W/cm^2](\log_{10})$) from isolated grafts. **(k)** IHC image from isolated graft for SCβ-cells (CPEP: c-peptide), scα-cells (GCG: glucagon) and scδ-cells (SST: somatostatin). (**l–n**) Endocrine hormone content. **(l)** Human insulin and **(m)** human glucagon content per graft mass (pmol/mg) *ex vivo*. **(n)** Human glucagon secretion in 1h as % of content in static assay in response to indicated secretagogues from hESC-derived endocrine cells (WT n=4, KO n=7) *in vitro*. All protein concentrations were measured by ELISA. All stem cell

differentiations were done for 27 days for *in vitro* assay and for transplantation. All grafts were isolated 30 weeks post-transplantation for *ex vivo* analysis. For scatter plots, each point in plots represents an independent biological experiment (n). Data are represented as mean ± SEM. Different letters designate significant differences within group. p-values: *$p<0.05$, **$p<0.01$, ***$p<0.001$; Mann-Whitney test. n.s: non-significant.

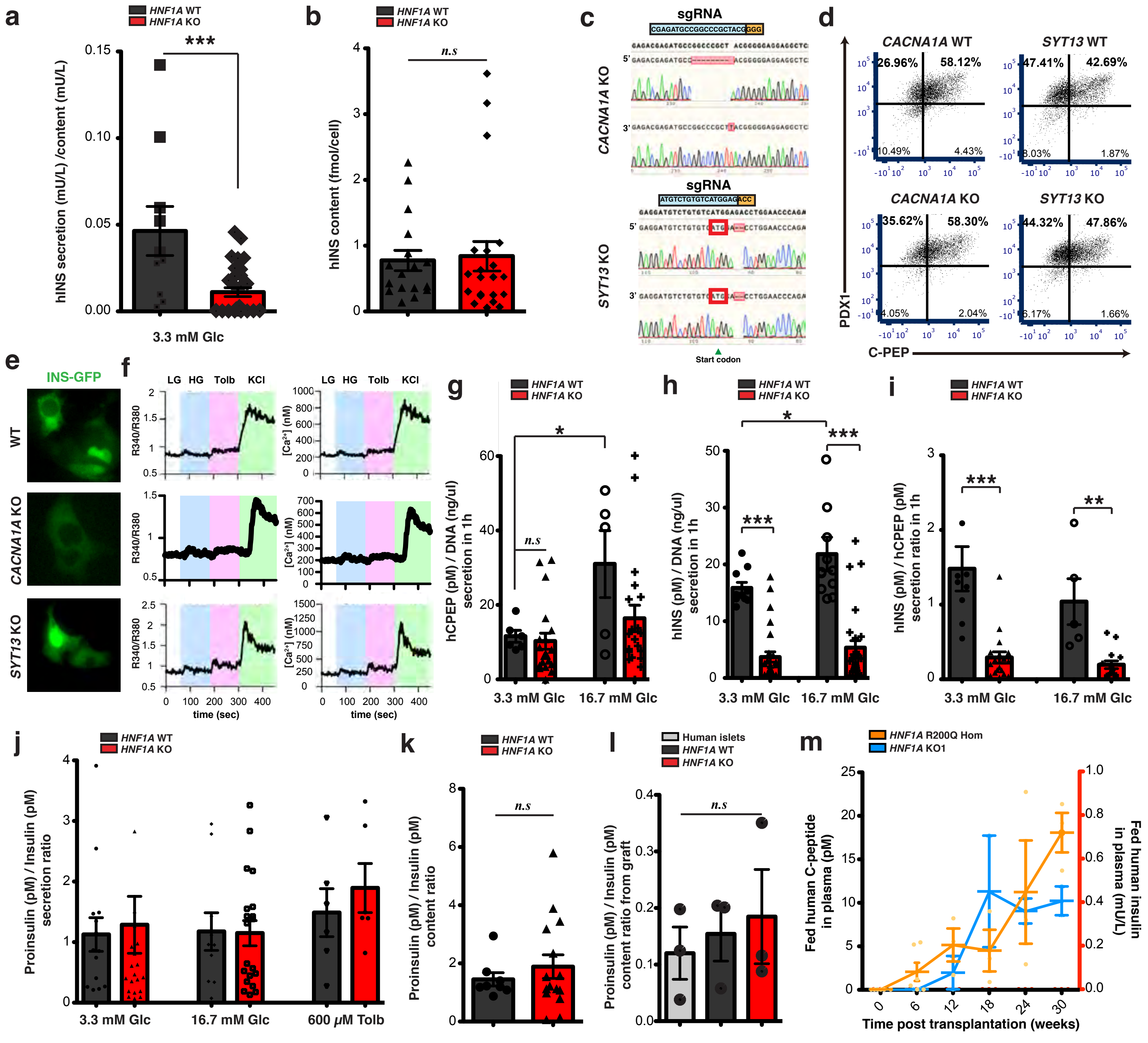

a
HNF1A WT
HNF1A KO
***
hINS secretion (mU/L) /content (mU/L)
3.3 mM Glc
b
n.s
hINS content (fmol/cell)
c
sgRNA
CACNA1A KO
SYT13 KO
Start codon
d
CACNA1A WT
SYT13 WT
CACNA1A KO
SYT13 KO
PDX1
C-PEP
e
INS-GFP
WT
f
LG HG Tolb KCl
R340/R380
[Ca2+] (nM)
time (sec)
g
hCPEP (pM) / DNA (ng/ul) secretion in 1h
3.3 mM Glc
16.7 mM Glc
h
hINS (pM) / DNA (ng/ul) secretion in 1h
i
hINS (pM) / hCPEP (pM) secretion ratio in 1h
j
Proinsulin (pM) / Insulin (pM) secretion ratio
600 μM Tolb
k
Proinsulin (pM) / Insulin (pM) content ratio
l
Human islets
Proinsulin (pM) / Insulin (pM) content ratio from graft
m
HNF1A R200Q Hom
HNF1A KO1
Fed human C-peptide in plasma (pM)
Fed human insulin in plasma (mU/L)
Time post transplantation (weeks)

Fig. S8. *HNF1A* deficiency affects insulin secretion in association with *CACNA1A* and *SYT13* down-regulation *in vitro*. (a) Human insulin secretion (mU/L) in 1h normalized to content (mU/L) in response to low glucose (LG, 3.3 mM) and high glucose (HG, 16.7 mM) stimulation in static assay. **(b)** Human insulin protein content per cell (fmol/cell) (WT n=17, KO n=21). **(c)** Sanger sequencing results showing knockout frameshift mutations in hESC *CACNA1A* KO and hESC *SYT13* KO line from both alleles near start codon (red box). **(d)** Representative flow cytometry of $CPEP^+$ and $PDX1^+$ populations. **(e)** dispersed scβ-like-cells (INS-GFP) with **(f)** representative intracellular calcium levels measured as R340/R380 and [Ca^{2+}](nM) over time (sec) with indicated secretagogues. **(g)** Human c-peptide (pM) and **(h)** human insulin secretion (pM) in 1h normalized to DNA (ng/µl) in response to basal (3.3 mM) and high (16.7 mM) glucose stimulation in static assay (WT n= 7 and KO n=20). **(i)** hINS(pM)/hCPEP(pM) secretion ratio in response to basal (3.3 mM) and high (16.7 mM) glucose stimulation (WT n=9 and KO n=14). **(j-l)** Proinsulin(pM)/Insulin(pM) ratio from **(j)** secretion and **(k)** content from *in vitro* cells (WT n=8 and KO n=15) and **(l)** *ex vivo* grafts. (**m**) Plasma human C-peptide (pM) (left y-axis) and human insulin secretion (mU/L) (right y-axis) in plasma of *ad libitum-fed* mice transplanted with hESC *HNF1A* KO1 and *HNF1A* R200Q homozygous-derived endocrine cells (n=3). All stem cell differentiations were done for 27 days. All protein concentrations were measured by ELISA. For scatter plots, each point in plots represents an independent biological experiment (n). Data are represented as mean ± SEM. p-values: *p<0.05, **p<0.01, ***p<0.001; Mann-Whitney test. n.s: non-significant.

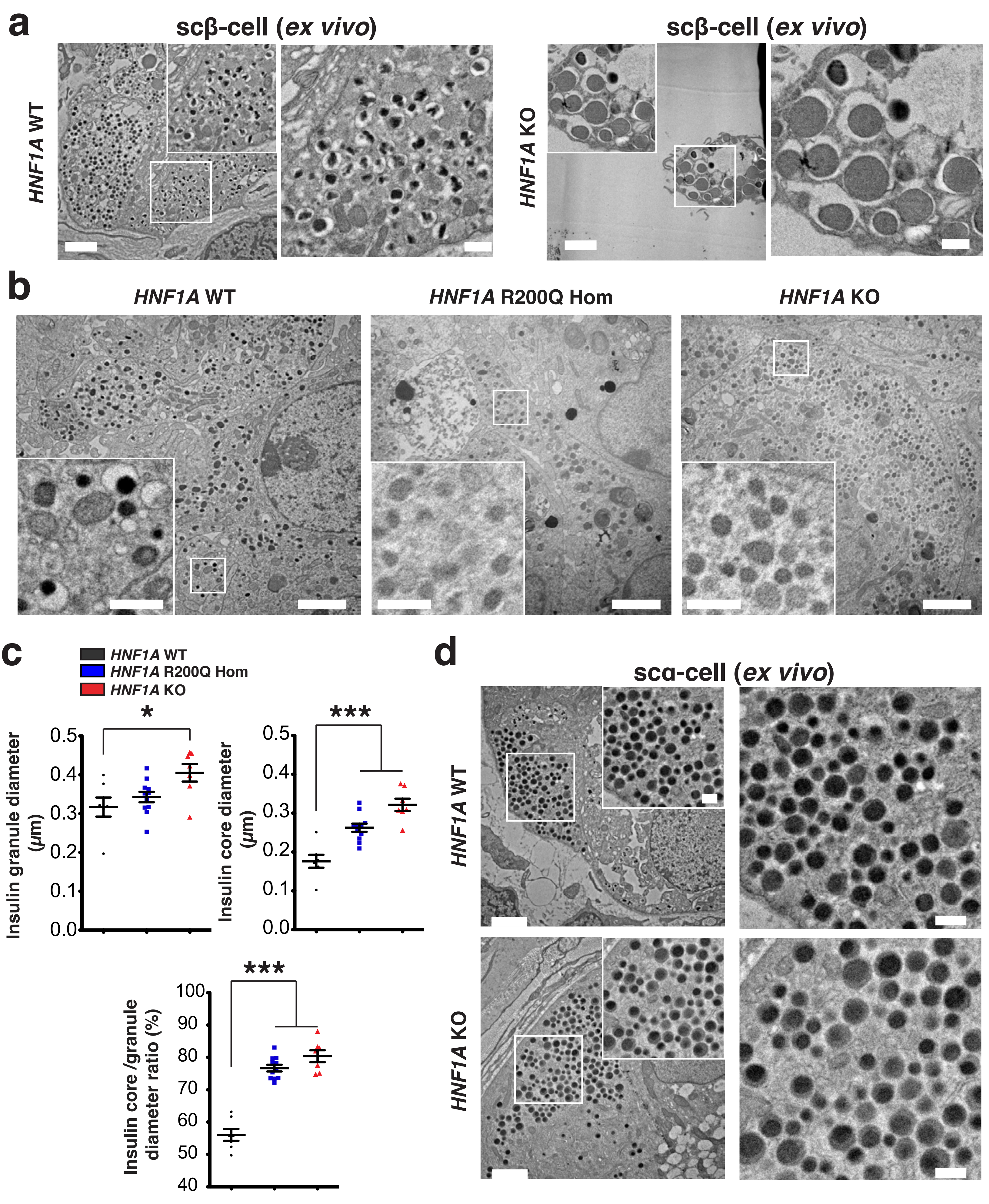

a
scβ-cell (ex vivo)
scβ-cell (ex vivo)
HNF1A WT
HNF1A KO
b
HNF1A WT
HNF1A R200Q Hom
HNF1A KO
c
HNF1A WT
HNF1A R200Q Hom
HNF1A KO
Insulin granule diameter (μm)
Insulin core diameter (μm)
Insulin core /granule diameter ratio (%)
d
scα-cell (ex vivo)
HNF1A WT
HNF1A KO

Fig. S9. *HNF1A* deficiency causes abnormal insulin granule structure. (**a**) Representative electron microscopy (EMC) image of scβ-cells *ex vivo* from isolated graft 30 weeks post-transplantation. Explants are from euglycemic mice. **(b)** Representative EMC images of scβ-like-cells *in vitro* with **(c)** quantification of insulin granule diameter (µm), insulin granule core diameter (µm) and insulin granule core diameter to insulin granule diameter ratio (%) per cell. **(d)** Representative EMC images of scα-cells. For all images, scale bars: 2 µm in low and 0.5 µm in high magnification. Each point in plots is the average of insulin granules per scβ-like-cells. All stem cell differentiations were done for 27 days for *in vitro* assay and for transplantation. For scatter plots, each point in plots represents an independent biological experiment (n). Data are represented as mean ± SEM. p-value: *p<0.05, **p<0.01, ***p<0.001; two-tailed t-test. n.s: non-significant.

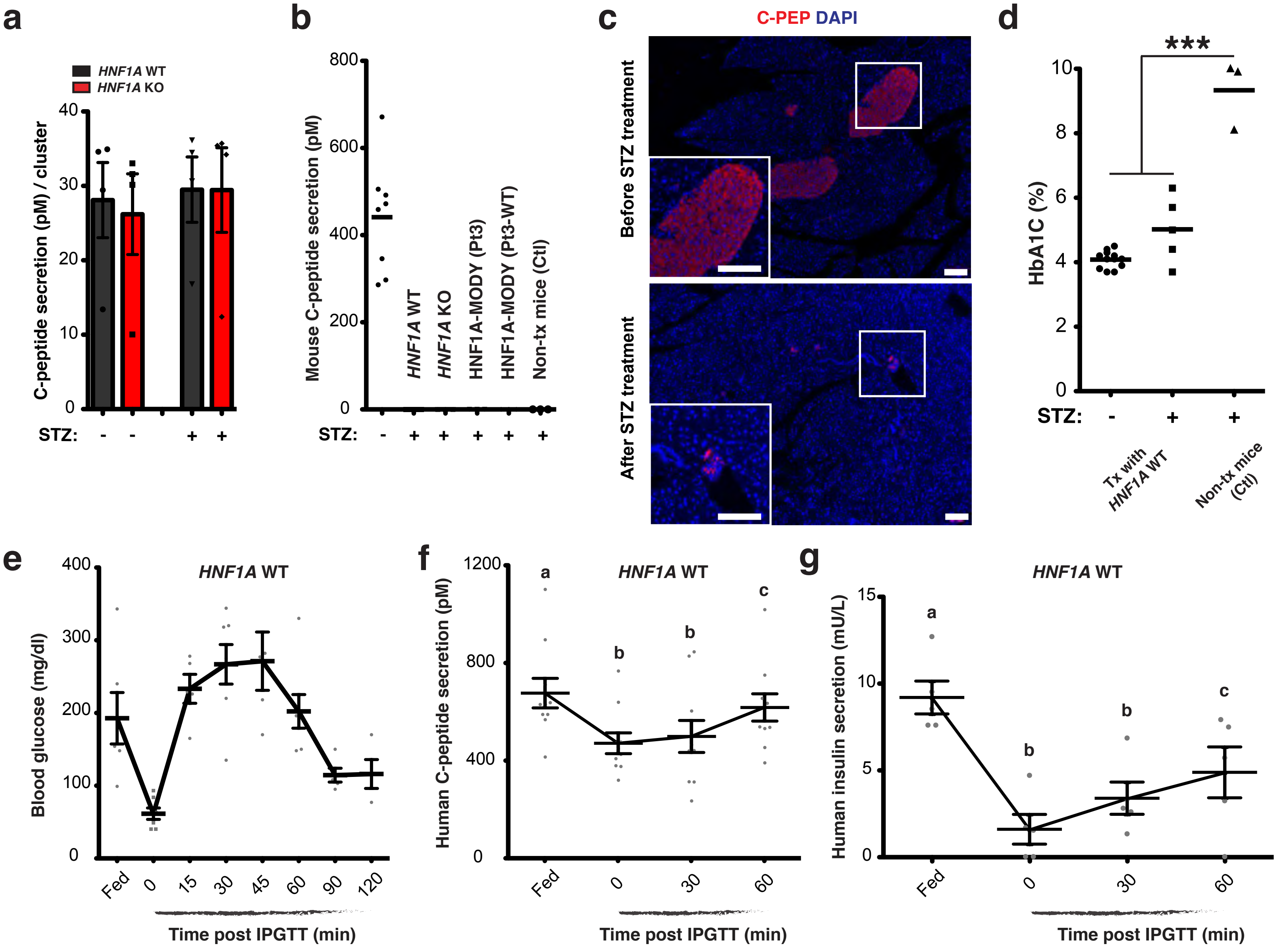

a
HNF1A WT
HNF1A KO
C-peptide secretion (pM) / cluster
40
30
20
10
0
STZ: - - + +
b
Mouse C-peptide secretion (pM)
800
600
400
200
0
HNF1A WT
HNF1A KO
HNF1A-MODY (Pt3)
HNF1A-MODY (Pt3-WT)
Non-tx mice (Ctl)
STZ: - + + + + +
c
C-PEP DAPI
Before STZ treatment
After STZ treatment
d
***
HbA1C (%)
10
8
6
4
2
0
STZ: - + +
Tx with HNF1A WT
Non-tx mice (Ctl)
e
HNF1A WT
Blood glucose (mg/dl)
400
300
200
100
0
Fed 0 15 30 45 60 90 120
Time post IPGTT (min)
f
HNF1A WT
Human C-peptide secretion (pM)
1200
800
400
0
a b b c
Fed 0 30 60
Time post IPGTT (min)
g
HNF1A WT
Human insulin secretion (mU/L)
15
10
5
0
a b b c
Fed 0 30 60
Time post IPGTT (min)

Fig. S10. *HNF1A* deficient scβ-cells are unable to maintain glucose homeostasis in diabetic mice. hESC-derived endocrine cells were treated with (4mg/ml; 15mM) or without STZ *in vitro*. **(a)** Human c-peptide secretion (pM) per cluster measured before and after STZ *in vitro*. **(b)** Mouse C-peptide secretion (pM) in plasma of *ad libitum-fed* mice transplanted with indicated cell lines or non-transplanted mice (Ctl) before (-) or >3 weeks after STZ injection (+). **(c)** Representative immunofluorescent staining of mouse pancreas showing islets (CPEP; red) before and after STZ treatment. Scale bars: 20 µm. **(d)** HbA1C (%) in mice transplanted with hESC *HNF1A* WT-derived endocrine cells or without cells (Ctl) before (-) and 5-15 weeks after (+) STZ treatment. (**e-g**) IPGTT in mice transplanted with hESC *HNF1A* WT-derived endocrine cells (n=6) several weeks (>3 weeks) after STZ treatment in *ad libitum-fed* state and during an iPGTT (t0, t30 and t60). **(e)** Blood glucose concentrations (mg/dl), **(f)** human C-peptide (pM) and **(g)** human insulin secretion (mU/L) in plasma. p-values were b: p<0.001, c: p<0.05. All stem cell differentiations were done for 27 days. For scatter plots, each point in plots represents an independent biological experiment (n). Data are represented as mean ± SEM. Different letters designate significant differences within group. p-values: *p<0.05, **p<0.01, ***p<0.001; two-tailed t-test.

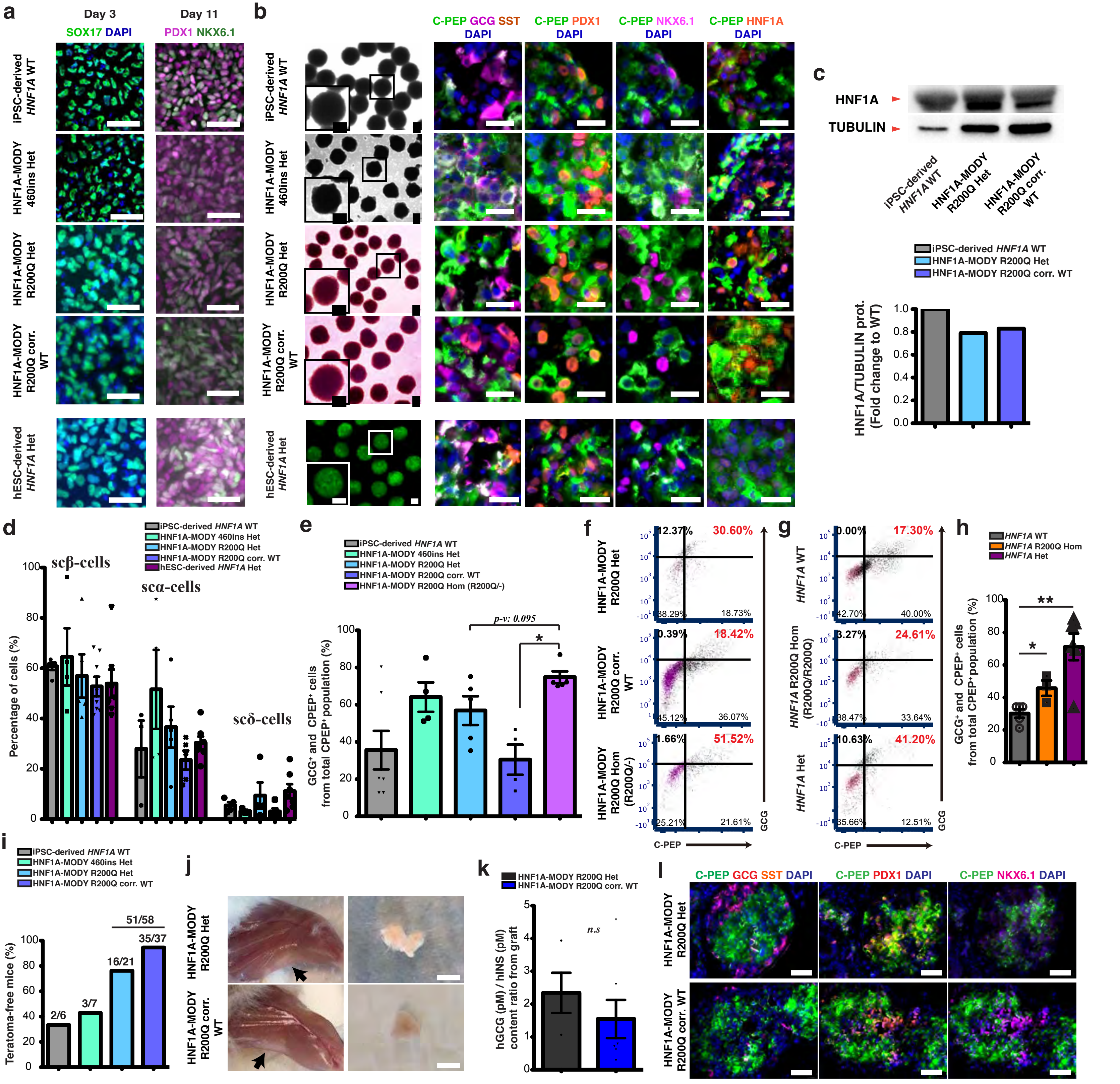

a
Day 3
SOX17 DAPI
Day 11
PDX1 NKX6.1
iPSC-derived HNF1A WT
HNF1A-MODY 460ins Het
HNF1A-MODY R200Q Het
HNF1A-MODY R200Q corr. WT
hESC-derived HNF1A Het
b
C-PEP GCG SST DAPI
C-PEP PDX1 DAPI
C-PEP NKX6.1 DAPI
C-PEP HNF1A DAPI
c
HNF1A
TUBULIN
iPSC-derived HNF1A WT
HNF1A-MODY R200Q Het
HNF1A-MODY R200Q corr. WT
HNF1A/TUBULIN prot. (Fold change to WT)
d
scβ-cells
scα-cells
scδ-cells
Percentage of cells (%)
hESC-derived HNF1A Het
e
HNF1A-MODY R200Q Hom (R200Q/-)
GCG+ and CPEP+ cells from total CPEP+ population (%)
p-v: 0.095
f
HNF1A-MODY R200Q Hom (R200Q/-)
12.37% 30.60% 38.29% 18.73%
0.39% 18.42% 45.12% 36.07%
1.66% 51.52% 25.21% 21.61%
C-PEP
GCG
g
HNF1A WT
HNF1A R200Q Hom (R200Q/R200Q)
HNF1A Het
0.00% 17.30% 42.70% 40.00%
3.27% 24.61% 38.47% 33.64%
10.63% 41.20% 35.66% 12.51%
h
HNF1A WT
HNF1A R200Q Hom
HNF1A Het
i
Teratoma-free mice (%)
2/6
3/7
16/21
35/37
51/58
j
k
HNF1A-MODY R200Q Het
HNF1A-MODY R200Q corr. WT
hGCG (pM) / hINS (pM) content ratio from graft
n.s
l
C-PEP GCG SST DAPI
C-PEP PDX1 DAPI
C-PEP NKX6.1 DAPI

Fig. S11. ***HNF1A*** **R200Q mutation is pathogenic and causes developmental bias towards the α-cell fate *in vitro*.** **(a)** Representative IHC staining of stem cell-derived cell lines *in vitro* at definitive endoderm stage with SOX17 (day 3), pancreatic progenitor stage with PDX1 and NKX6.1 (day 11) and endocrine cell stage (day 27). Scale bars: 50 µm. White cells are PDX1/NKX6.1 double positive cells. **(b)** Representative bright-field images from HNF1A-MODY iPSC-derived organoids and GFP image from hESC Het-derived organoids with IHC staining for indicated markers. White cells are GCG/CPEP double positive cells. Scale bars: 20 µm. **(c)** Western blot for HNF1A (67 kDa) and β-Tubulin-III (55 kDa) from iPSC-derived endocrine cells with respective quantification on day 27 of differentiation *in vitro*. **(d)** Percentage of scβ-like-cells ($CPEP^{+}$/$PDX1^{+}$), scα-like-cells (GCG^{+}) and scδ-like-cells (SST^{+}) determined by IHC. **(e)** Quantification of GCG and CPEP positive cells from total CPEP positive cell population determined by IHC. **(f)** $CPEP^{+}$ and GCG^{+} populations from iPSC-derived endocrine cells and **(g)** hESC-derived endocrine ells by flow cytometry. Gating for GCG and CPEP negative cells (magenta) was determined by incubating cells without primary antibodies and with secondary antibodies. **(h)** Quantification of GCG and CPEP positive cells from total CPEP positive cell population (%) determined by flow cytometry. **(i)** Teratoma-free mice transplanted with HNF1A-MODY iPSC-derived endocrine cells. Graft tissue was considered a teratoma when larger than 2 cm and by IHC of the germ layers. **(j)** Representative image of graft tissue (black arrows) 30 weeks post-transplantation with HNF1A-MODY iPSC-derived endocrine cells before and after explant from quadriceps muscle. Scale bars: 0.5 cm. **(k)** Human glucagon content per graft mass (pmol/mg) *ex vivo* measured by ELISA. **(l)** Representative IHC images showing HNF1A-MODY iPSC-derived endocrine cells from isolated graft. Scale: 50 µm. All stem cell differentiations were

done for 27 days for *in vitro* assay and for transplantation. All grafts were isolated 30 weeks post-transplantation for *ex vivo* analysis. For scatter plots, each point in plots represents an independent biological experiment (n). Data are represented as mean ± SEM. Different letters designate significant differences within group. p-values: *$p<0.05$, **$p<0.01$, ***$p<0.001$; Mann-Whitney test. n.s: non-significant.

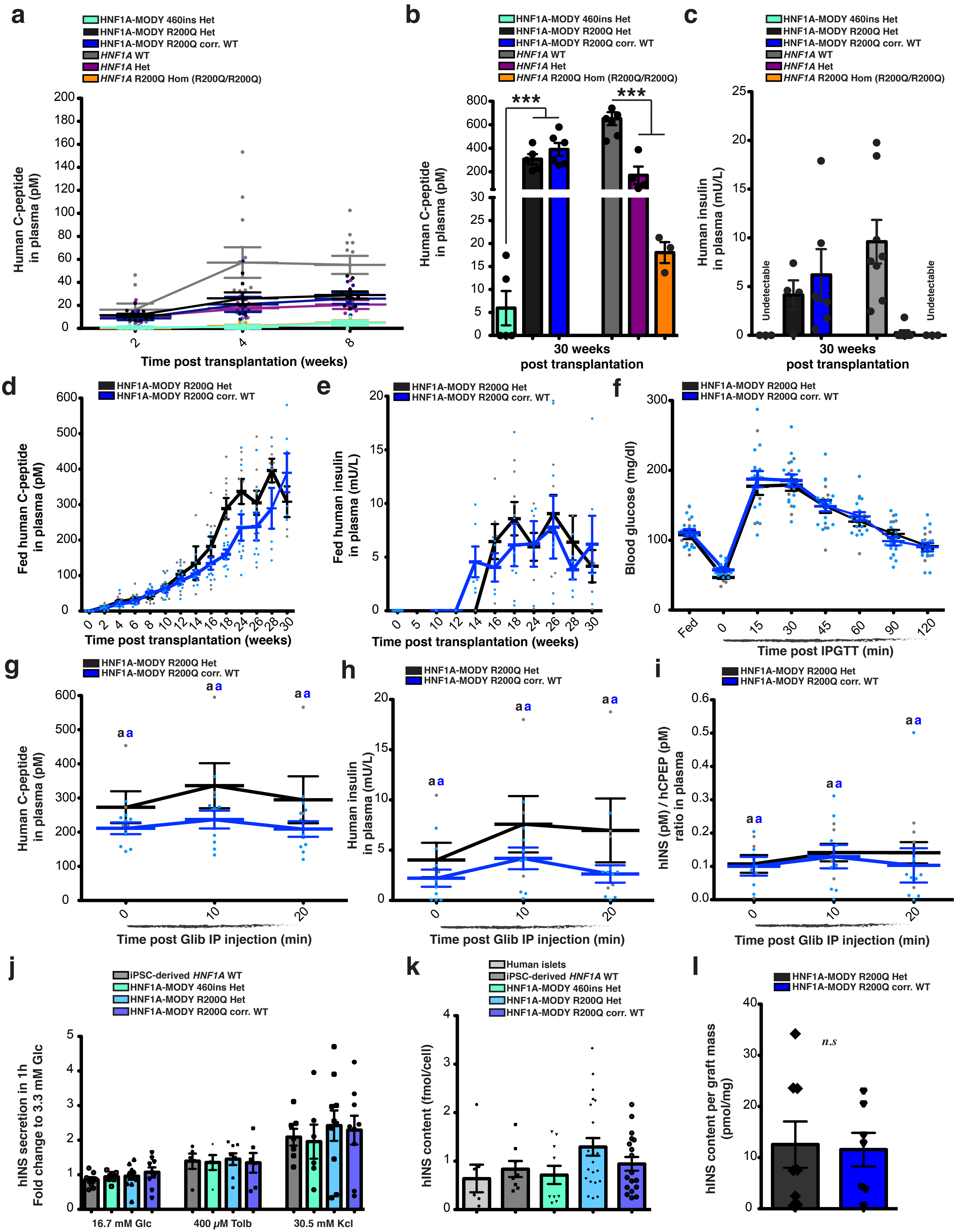
a
HNF1A-MODY 460ins Het
HNF1A-MODY R200Q Het
HNF1A-MODY R200Q corr. WT
HNF1A WT
HNF1A Het
HNF1A R200Q Hom (R200Q/R200Q)
Human C-peptide in plasma (pM)
Time post transplantation (weeks)
b
***
***
Human C-peptide in plasma (pM)
30 weeks post transplantation
c
Human insulin in plasma (mU/L)
Undetectable
Undetectable
30 weeks post transplantation
d
HNF1A-MODY R200Q Het
HNF1A-MODY R200Q corr. WT
Fed human C-peptide in plasma (pM)
Time post transplantation (weeks)
e
Fed human insulin in plasma (mU/L)
Time post transplantation (weeks)
f
Blood glucose (mg/dl)
Fed
Time post IPGTT (min)
g
Human C-peptide in plasma (pM)
a a
Time post Glib IP injection (min)
h
Human insulin in plasma (mU/L)
Time post Glib IP injection (min)
i
hINS (pM) / hCPEP (pM) ratio in plasma
Time post Glib IP injection (min)
j
iPSC-derived HNF1A WT
HNF1A-MODY 460ins Het
HNF1A-MODY R200Q Het
HNF1A-MODY R200Q corr. WT
hINS secretion in 1h Fold change to 3.3 mM Glc
16.7 mM Glc
400 μM Tolb
30.5 mM Kcl
k
Human islets
iPSC-derived HNF1A WT
HNF1A-MODY 460ins Het
HNF1A-MODY R200Q Het
HNF1A-MODY R200Q corr. WT
hINS content (fmol/cell)
l
HNF1A-MODY R200Q Het
HNF1A-MODY R200Q corr. WT
n.s
hINS content per graft mass (pmol/mg)

Fig. S12. HNF1A-MODY iPSC-derived β-cells are initially glucose responsive. (**a-c**) Human C-peptide (pM) and human insulin levels in plasma of *ad libitum-fed* mice transplanted with HNF1A-MODY iPSC- and hESC-derived endocrine cells**.** **(a)** Plasma human C-peptide (pM) two, four and eight weeks post transplantation in mice transplanted with HNF1A-MODY iPSC lines (460ins Het n=4, R200Q Het n=12 and R200Q corr. WT n=12) and hESC lines (WT n=17, Het n=10 and R200Q Hom n=6). **(b)** Plasma human C-peptide (pM) thirty weeks post transplantation in mice transplanted with HNF1A-MODY iPSC lines (460ins Het n= 5, R200Q Het n=5 and R200Q corr. WT n=6) and hESC lines (WT n=7, Het n=4 and R200Q Hom n=3) and **(c)** human insulin (mU/L) thirty weeks post transplantation in mice transplanted with HNF1A-MODY iPSC lines (460ins Het n= 3, R200Q Het n=4 and R200Q corr. WT n=6) and hESC lines (WT n=8, Het n=7 and R200Q Hom n=3). **(d)** Plasma human C-peptide (pM) and **(e)** human insulin secretion (mU/L) in plasma of *ad libitum-fed* mice transplanted with HNF1A-MODY iPSC-derived endocrine cells (R200Q Het n=14 and R200Q corr. WT n=12). **(f)** Blood glucose concentrations (mg/dl) in *ad libitum-fed* state and during an iPGTT (t0, t30 and t60) in mice with HNF1A-MODY iPSC-derived endocrine cells (R200Q Het n=12 and R200Q corr. WT n=10). **(g-i)** Glibenclamide injection in *ad libitum-fed* mice transplanted with HNF1A-MODY iPSC-derived endocrine cells (R200Q Het n=5 and R200Q corr. WT n=9). **(g)** Human C-peptide secretion (pM), **(h)** human insulin secretion (mU/L) and **(i)** hINS (pM)/hCPEP (pM) secretion ratios. **(j)** Human insulin secretion in 1h from static assay as a fold change to 3.3 mM Glc in response to indicated secretagogues *in vitro* (iPSC WT n=5, 460ins Het n=5, R200Q Het n=10 and R200Q corr. WT n=7). **(k)** Human insulin protein content per cell (fmol/cell) *in vitro* (Human islets n=6, iPSC WT n=4, 460ins Het n=4, R200Q Het n=7 and R200Q corr. WT n=5). **(l)** Human insulin content per graft mass (pmol/mg). All stem cell differentiations were done for 27 days for *in vitro* assay and

for transplantation. All grafts were isolated 30 weeks post-transplantation for *ex vivo* analysis. All protein concentrations were measured by ELISA. For scatter plots, each point in plots represents an independent biological experiment (n). Data are represented as mean ± SEM. Different letters designate significant differences within group. p-values: *$p<0.05$, **$p<0.01$, ***$p<0.001$; two-tailed t-test. n.s: non-significant.

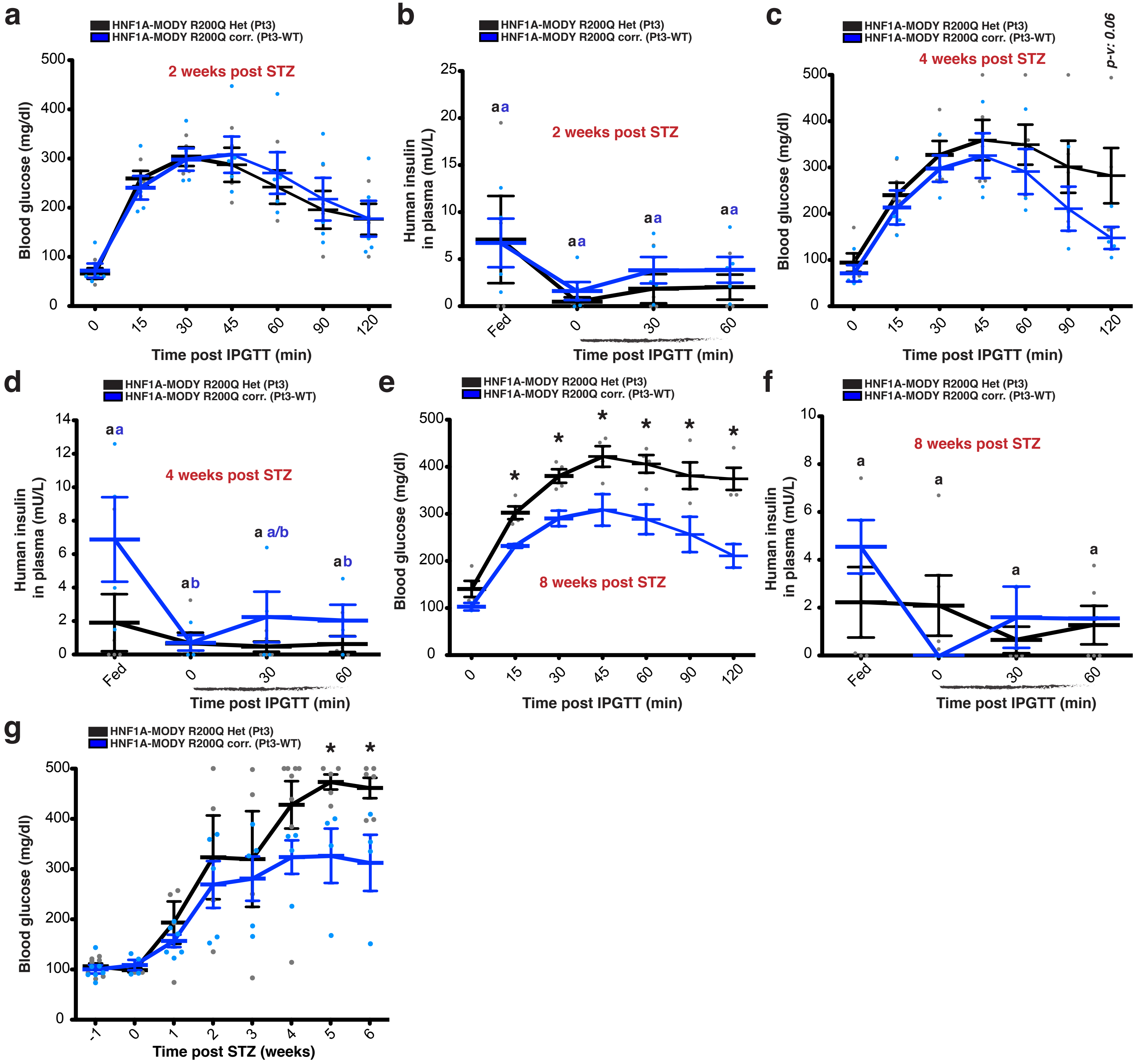

a
HNF1A-MODY R200Q Het (Pt3)
HNF1A-MODY R200Q corr. (Pt3-WT)
2 weeks post STZ
Blood glucose (mg/dl)
Time post IPGTT (min)
b
Human insulin in plasma (mU/L)
c
4 weeks post STZ
p-v: 0.06
d
e
8 weeks post STZ
f
g
Time post STZ (weeks)

Fig. S13. *HNF1A* haploinsufficiency gradually impairs scβ-cell function *in vivo.* (**a-f**) IPGTT in mice transplanted with HNF1A-MODY iPSC-derived endocrine cells in *ad libitum-fed* state and during an iPGTT (t0, t30 and t60) 2, 4 and 8 weeks post STZ treatment. **(a)** Blood glucose concentrations (mg/dl) and **(b)** human insulin secretion (mU/L) in plasma of mice after 2 weeks post STZ (R200Q Het n=12; R200Q corr. WT n=10). **(c)** Blood glucose concentrations (mg/dl) and **(d)** human insulin secretion (mU/L) in plasma of mice after 4 weeks post STZ (R200Q Het n=5; R200Q corr. WT n=4). **(e)** Blood glucose concentrations (mg/dl) and **(f)** human insulin secretion (mU/L) in plasma of mice after 8 weeks post STZ (R200Q Het n=5; R200Q corr. WT n=2). p-values were b and c: $p<0.05$. **(g)** Blood glucose levels (mg/dl) monitored in *ad libitum-fed* mice weeks before (-1) or after (0 to 6) STZ injection (R200Q Het n=10; R200Q corr. Wt n=7). All protein concentrations were measured by ELISA. For scatter plots, each point in plots represents an independent biological experiment (n). Data are represented as mean ± SEM. Different letters designate significant differences between fed, t0, t30 and t60 for each genotype. p-values: *$p<0.05$, **$p<0.01$, ***$p<0.001$; two-tailed t-test.

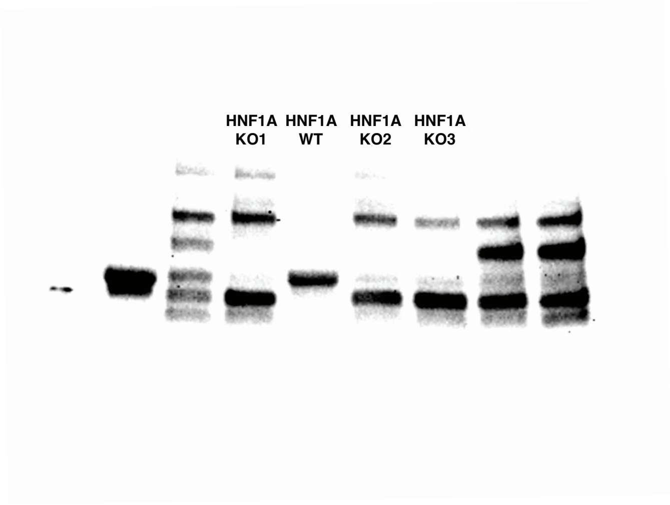
HNF1A KO1
HNF1A WT
HNF1A KO2
HNF1A KO3

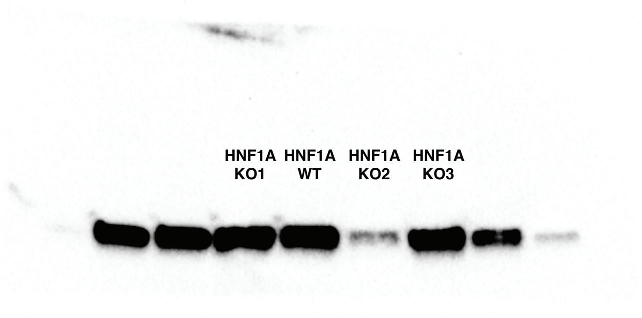
HNF1A KO1
HNF1A WT
HNF1A KO2
HNF1A KO3

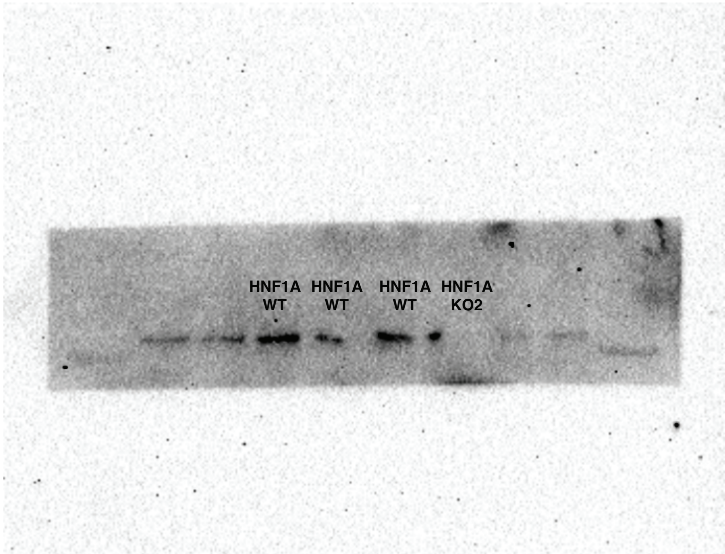
HNF1A WT
HNF1A WT
HNF1A WT
HNF1A KO2

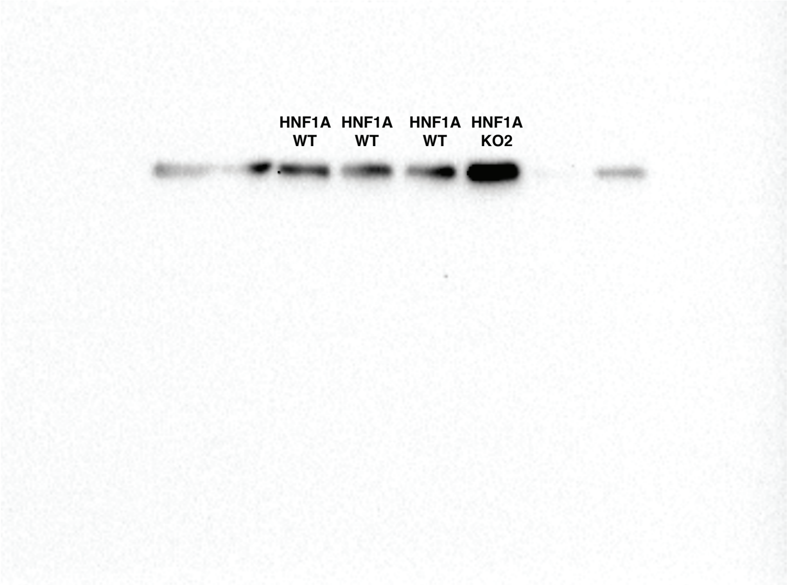
HNF1A WT
HNF1A WT
HNF1A WT
HNF1A KO2

Fig. S14. Western Blot raw images.
